# Lymph node fibroblastic reticular cells preserve a tolerogenic niche in allograft transplantation through laminin **α**4

**DOI:** 10.1172/JCI156994

**Published:** 2022-07-01

**Authors:** Lushen Li, Marina W. Shirkey, Tianshu Zhang, Wenji Piao, Xiaofei Li, Jing Zhao, Zhongcheng Mei, Yizhan Guo, Vikas Saxena, Allison Kensiski, Samuel J. Gavzy, Yang Song, Bing Ma, Jing Wu, Yanbao Xiong, Long Wu, Xiaoxuan Fan, Holly Roussey, Meng Li, Alexæander S. Krupnick, Reza Abdi, Jonathan S. Bromberg

**Affiliations:** 1 Department of Surgery and; 2 Center for Vascular and Inflammatory Diseases, University of Maryland School of Medicine, Baltimore, Maryland, USA.; 3 Transplantation Research Center, Renal Division, Brigham and Women’s Hospital, Harvard Medical School, Boston, Massachusetts, USA.; 4 Institute for Genome Sciences,; 5 Flow Cytometry Shared Service, Greenebaum Comprehensive Cancer Center. and; 6 Institute of Human Virology, University of Maryland School of Medicine, Baltimore, Maryland, USA.

**Keywords:** Cell Biology, Transplantation, Adaptive immunity, Laminin, T cell development

## Abstract

Lymph node (LN) fibroblastic reticular cells (FRCs) define LN niches and regulate lymphocyte homeostasis through producing diverse extracellular matrix (ECM) components. We examined the role of ECM laminin α4 (Lama4) using FRC-Lama4 conditional KO *Pdgfrb-Cre^–/–^* × *Lama4^fl/fl^* mice. Single-cell RNA-sequencing (scRNA-Seq) data showed the promoter gene *Pdgfrb* was exclusively expressed in FRCs. Depleting FRC-Lama4 reduced Tregs and dendritic cells, decreased high endothelial venules, impaired the conduit system, and downregulated T cell survival factors in LNs. FRC-Lama4 depletion impaired the homing of lymphocytes to LNs in homeostasis and after allografting. Alloantigen-specific T cells proliferated, were activated to greater degrees in LNs lacking FRC-Lama4, and were more prone to differentiate into effector phenotypes relative to the Treg phenotype. In murine cardiac transplantation, tolerogenic immunosuppression was not effective in FRC-Lama4 recipients, which produced more alloantibodies than WT. After lung transplantation, FRC-Lama4–KO mice had more severe graft rejection with fewer Tregs in their LNs. Overall, FRC-Lama4 critically contributes to a tolerogenic LN niche by supporting T cell migration, constraining T cell activation and proliferation, and promoting Treg differentiation. Hence, it serves as a therapeutic target for immunoengineering.

## Graphical abstract

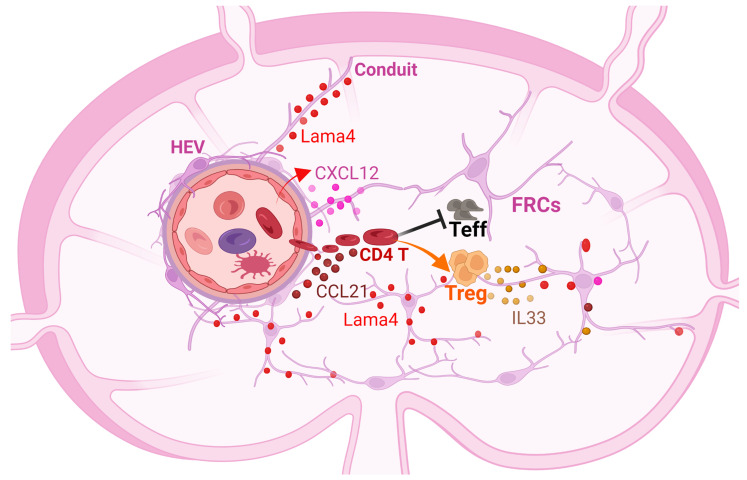

## Introduction

Mammalian lymph nodes (LNs) serve as the primary initiation sites of immune responses, acting by choreographing the interactions of T cells, B cells, macrophages, and dendritic cell (DCs). The sophisticated 3D LN architecture is underpinned by nonhematopoietic LN stromal cells (LNSCs), including blood endothelial cells (BECs) (Pdpn^–^CD31^+^, where Pdpn indicates podoplanin), lymphatic endothelial cells (LECs) (Pdpn^+^CD31^+^),and fibroblastic reticular cells (FRCs) (Pdpn^+^CD31^–^) (1–3). In human and mouse FRCs, platelet-derived growth factor receptor-β (Pdgfrβ) gene expression is highly associated with other markers, such as *CCL19*, and *CCL21* (4). The Pdgfrβ protein is widely distributed in LN regions, including medulla, interfollicular area, and T cell zone (T-zone) (5). FRCs support LN architecture and vasculature by producing various extracellular matrix (ECM) components, including collagens, ER-TR7, fibronectin, and laminins, which construct structural scaffolds for immune cell interactions (6). FRCs also support endothelial cell proliferation and vasculature development through secreting VEGF (7).The plentiful vascular structures contribute to immune responses by supporting immune surveillance, cell and molecule exchange, and antigen recognition. Blood-borne leukocytes enter LNs via high endothelial venules (HEVs) and subsequently migrate to the B cell zone or T-zone to encounter cognate antigens (8).

The LNs are important sites of immune regulation. FRCs support T cell migration, proliferation, survival, and differentiation by secreting numerous soluble factors, including delta-like 4 (DLL4), CXCL12, CCL19, CCL21, IL-6, IL-7, IL-15, IL-33, and CD40 (9–11). IL-33 supports Tregs that control graft rejection and prevent graft‑versus‑host disease (GVHD) in transplantation (12). Administration of IL-33 increases Treg frequency with enhanced suppressive activity and, in turn, promotes long-term allograft survival in murine skin and heart transplantations (12, 13). Mouse and human FRCs limit T effector functions through nitric oxide (NO) or constitutive cyclooxygenase (COX) enzymes (9, 14). The critical functions of FRCs suggest promising FRC-based therapeutic strategies to maintain immune homeostasis, trigger effective immune responses, and improve transplant tolerance. For instance, following cardiac transplantation, donor mast cells trigger FRC senescence and upregulate collagen I deposition, thereby forming a proinflammatory niche in draining LNs (dLNs). Consequently, the LNs are not restored to a homeostatic state, but retain more severe fibrosis following organ rejection (15). Such an effect is referred to as “immunological scarring” (16) and is ameliorated by administrating ex vivo–expanded FRCs to the recipients, which also improves allograft survival (15).

Laminin expression is dynamically associated with tolerance and immunity. Differential expression of laminin α4 (Lama4) and α5 (Lama5) in the LN cortical ridge (CR) and the basement of HEVs is correlated with tolerance and immunity, respectively (17). We previously evaluated laminin expression in diverse immune regimens, including immune stimulation with CFA, alloantigen stimulation with donor-specific splenocyte transfusion (DST), tolerance induction with DST plus anti-CD40L mAbs, and immune suppression with a progressively growing tumor. In each case, a decreased Lama4/Lama5 ratio correlates with immunity and inflammation, while an increased ratio correlates with tolerance and suppression (18). In vitro analyses showed that laminin 411 and 511 proteins regulate T helper cell migration, proliferation, and differentiation and Treg inductions (18). LN stromal Lama5 alters immune responses through LN structures and T cell functions (19). The specific role of FRC-derived Lama4 remains elusive.

Here, we verified the critical role of FRCs in regulating LN structure, cell abundance, and cytokine/chemokine production using Ccl19-Cre–inducible diphtheria toxin (DT) receptor (CCL19/iDTR) mice. We further elaborated the role of FRC-derived Lama4 by creating an FRC-Lama4 conditional KO mouse model (*Pdgfrb-Cre^–/–^ × Lama4^fl/fl^*). Depleting FRC-derived Lama4 remodeled the infrastructure for immune cell homing. It also resulted in a dedicated niche that promoted T cell adaptive immunity and altered T effector cell differentiation in response to alloantigens. Consequently, ablation of FRC-Lama4 impaired immune suppression and allograft survival in murine lung and cardiac allograft transplantation. Collectively, the present study revealed the critical roles of LN FRCs and the special FRC-Lama4 in regulating allogeneic responses.

## Results

### FRC depletion impairs LN structure and cellularity.

**Figure 1:**
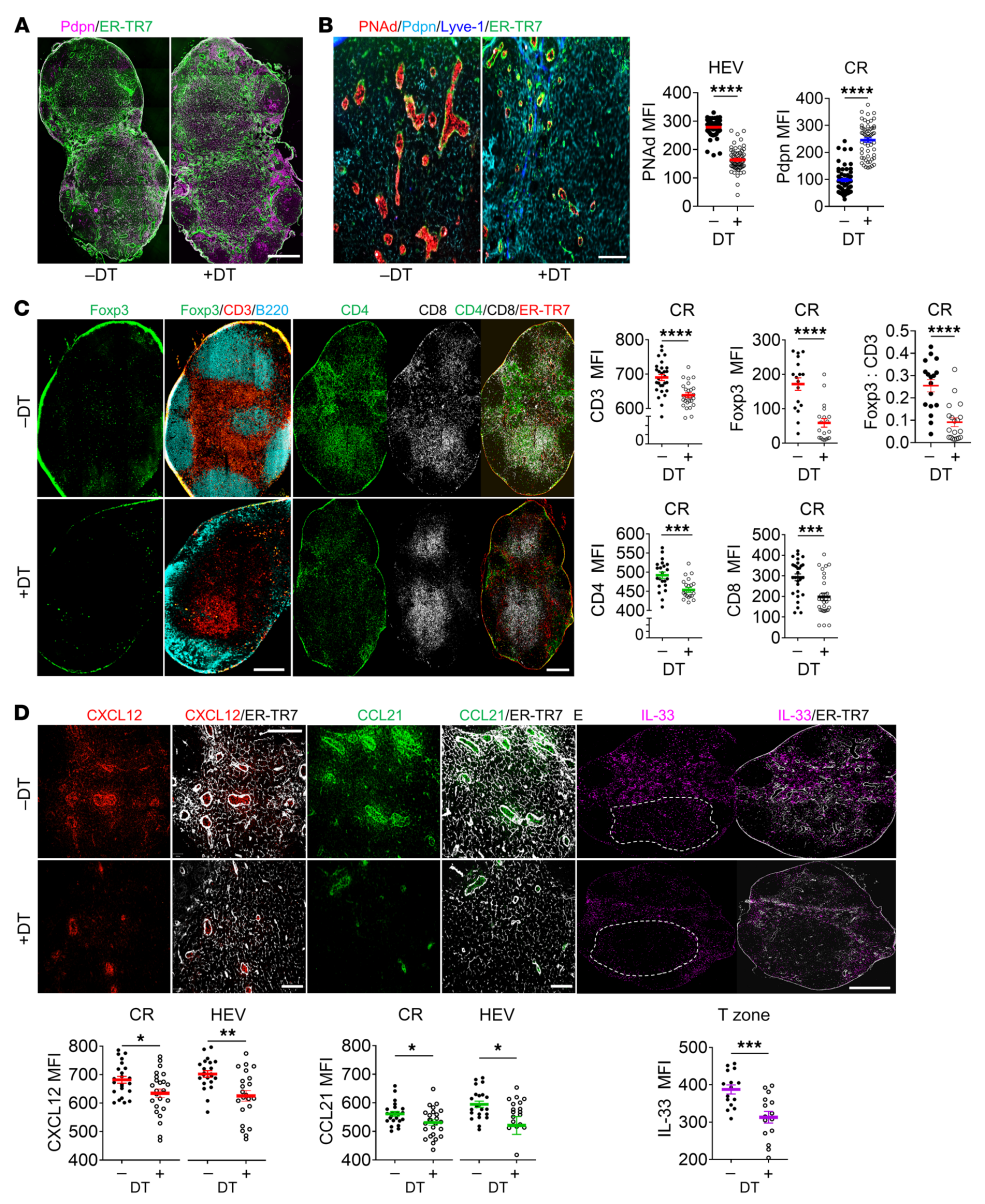
FRCs support LN structure and cellularity. To deplete FRCs, CCL19/iDTR mice were treated with DT (i.p. 100 ng/d × 5 days). (**A** and **B**) Whole-mount scanning (**A**) and fluorescent images (**B**) of LN cryosections stained for ER-TR7, Pdpn, PNAd, and Lyve-1. Original magnification, ×20. Scale bars: 500 μm (**A**); 100 μm (**B**). Quantification of PNAd intensity in HEV areas and Pdpn intensity in CR. (**C**) Whole-mount scanning of LN cryosections stained for Foxp3, CD3, B220, CD4, CD8, and ER-TR7. Original magnification, ×20. Scale bars: 500 μm. Quantification of CD3, Foxp3, CD4, and CD8 intensity, and Foxp3/CD3 ratio in CR. (**D**) Representative fluorescent images of LN cryosections stained for CXCL12, CCL21, and ER-TR7. Scale bars: 100 μm. Quantification of intensity in CR and around HEVs. (**E**) Whole-mount scanning of LN cryosections stained for IL-33 and ER-TR7. Scale bar: 500 μm. Quantification of intensity in T-zone. (**A**–**E**) Three independent experiments, 3 mice/group, 3 LNs/mouse, 3 sections/LN, 3 to 5 fields/section. Student’s unpaired, 2-tailed *t* test for 2-group comparisons. Data are represented as mean ± SEM. *P < 0.05; **P < 0.01; ***P < 0.001; ****P < 0.0001. *P* < 0.05 was considered significant.

To verify the contribution of FRCs to LN structure and cellularity, we assessed DT-inducible FRC depletion in LNs from CCL19/iDTR mice. Administration of DT caused a dramatic reduction of FRC frequency in CCL19/iDTR mice, without affecting BECs and LECs (Supplemental Figure 1; supplemental material available online with this article; https://doi.org/10.1172/JCI156994DS1). Consequently, depleting FRCs resulted in a collapsed FRC network, as indicated by the poorly organized ER-TR7^+^ fibroblastic network (Figure 1A)” and caused the impairment of the CD3^+^ T-zone and B220^+^ B follicles (Figure 1C). Compared with non-DT-treated controls, depleting FRCs resulted in fewer and smaller HEVs with decreased expression of peripheral node addressin (PNAd), the hallmark of postcapillary vessel maturation into highly differentiated HEVs (ref. 20 and Figure 1B). Abundance of Foxp3^+^ Tregs and CD3^+^, CD4^+^, and CD8^+^ T cells all decreased in the CR (Figure 1C). The Tregs decreased even more dramatically relative to total CD3^+^ T cells, as evidenced by the decreased Foxp3/CD3 ratio (Figure 1C), indicating that Tregs were more sensitive to FRC depletion relative to other T cell subtypes. Since migration of these cells from blood to LNs is driven by the chemokines CCL21 and CXCL12, we assessed these chemokines in CCL19/iDTR mouse LNs. FRC depletion decreased CXCL12 and CCL21 in the CR and around HEVs (Figure 1D). IL-33, an FRC-derived cytokine with pleiotropic properties and supportive effects on Tregs and type 2 innate immune cells (ILC2s) (12), was decreased in the deep T-zone (Figure 1E), a domain normally enriched with CCL19^+^ FRCs (5). Collectively, these results demonstrate that FRCs support LN architecture and HEV maturation and secrete chemokines and cytokines required for lymphocyte migration and survival in LNs.

### FRC-Lama4 depletion reduces LN homeostatic Tregs and DCs.

**Figure 2:**
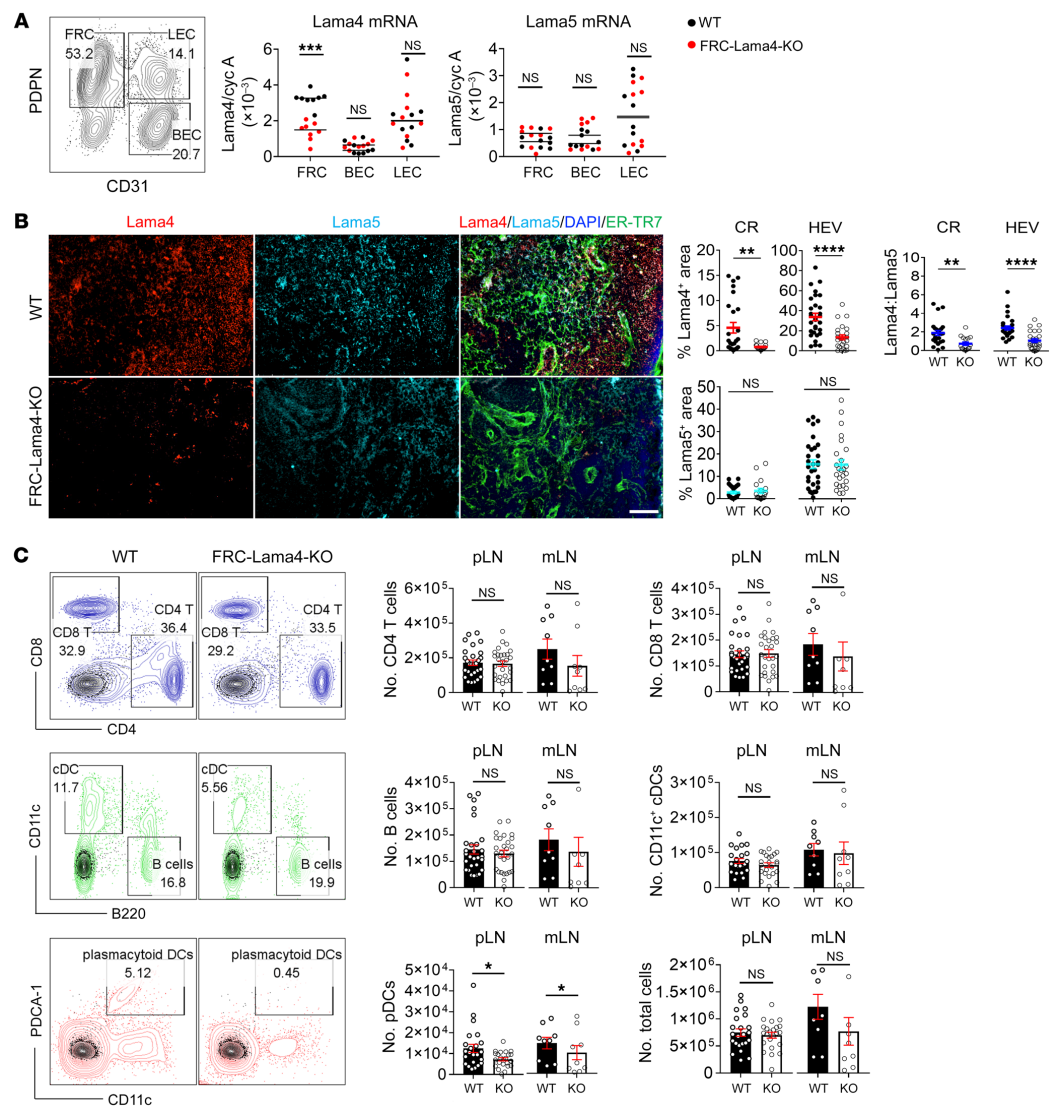
FRC-Lama4 conditional KO mouse construction and characterization. (**A**) Gating strategy for sorting FRCs, BECs, and LECs (left); values show percentages. Lama4 and Lama5 mRNA relative to cyclophilin A in FRCs, BECs, and LECs from FRC-Lama4–KO and littermate control (WT) LNs (right, qRT-PCR). (**B**) Lama4 and Lama5 protein in FRC-Lama4–KO and WT LNs. Representative images of LN cryosections stained for Lama4, Lama5, and ER-TR7. Original magnification, ×20. Scale bar: 100 μm. Quantification of Lama4- and Lama5-positive area percentages and Lama4/Lama5 ratios in the CR and around HEV. (**C**) Cellularity in FRC-Lama4–KO and WT LNs. Left: gating strategies for CD4^+^ T cells, CD8^+^ T cells, B cells, cDCs, and pDCs in WT LNs; values show percentage. Right: number of cells in each LN. Three independent experiments, 3 mice/group, 3 LNs/mouse, 3 sections/LN, and 3 to 5 fields/section. Student’s unpaired 2-tailed *t* test for 2-group comparisons. Data are represented as mean ± SEM. *P < 0.05; **P < 0.01; ***P < 0.001; ****P < 0.0001. *P* < 0.05 was considered significant.

**Figure 3:**
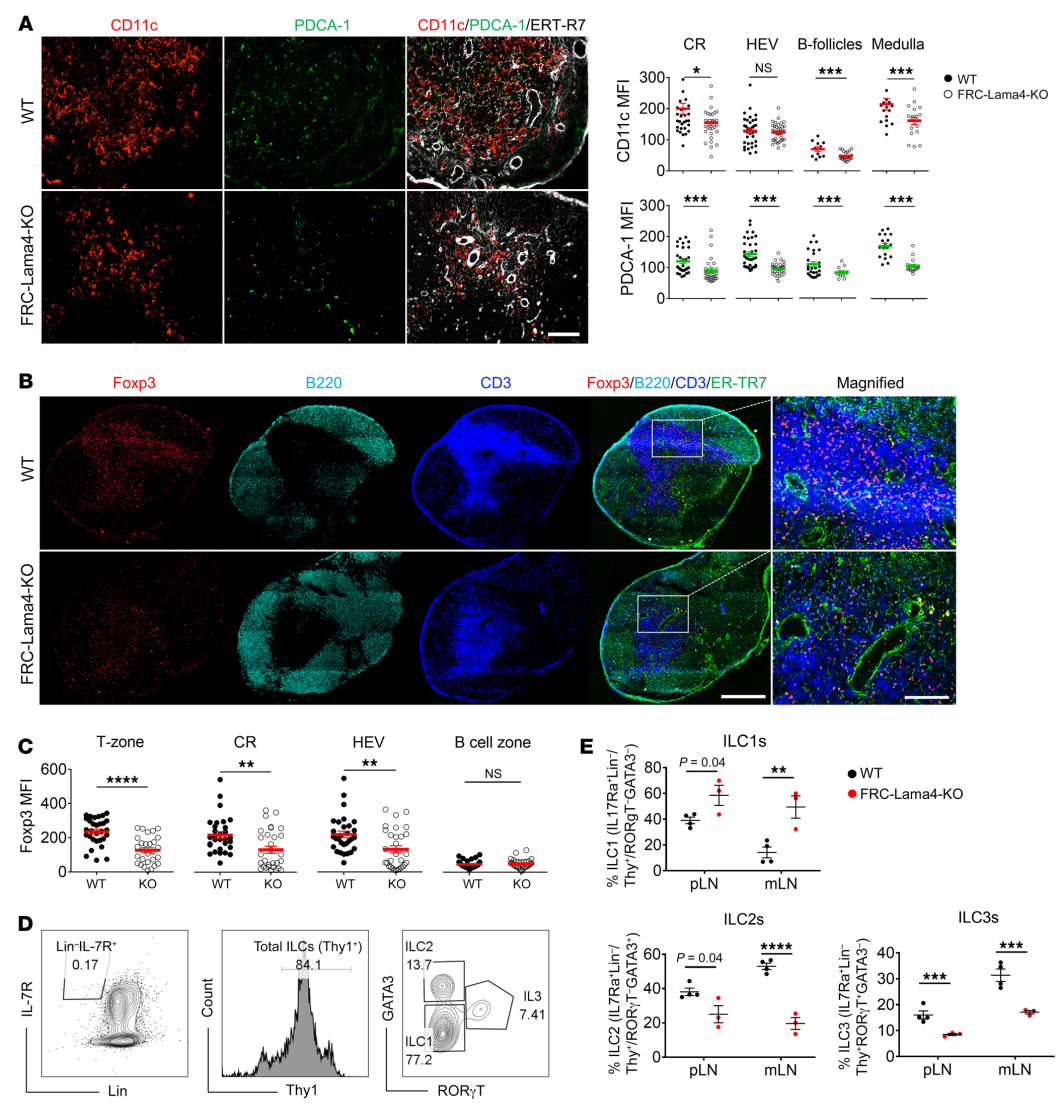
FRC-Lama4 depletion coincides with decreased DCs and Tregs and altered ILCs. (**A**) Fluorescent images of FRC-Lama4–KO and WT LN cryosections stained for CD11c, PDCA1, and ER-TR7. Original magnification, ×20. Scale bar: 100 μm. Quantification of CD11c and PDCA-1 intensity in indicated LN regions. (**B**) Whole-mount scanning of LN cryosections from FRC-Lama4–KO and WT mice, stained for Foxp3, B220, CD3, and ER-TR7. Original magnification, ×20. Scale bars: 500 μm (left); 100 μm (right). (**C**) Quantification of Foxp3 intensity in indicated LN regions. (**D** and **E**) Gating strategy (**D**) (values show percentages) and quantification (**E**) of ILC subsets in LNs. Representative of 3 independent experiments. (**A**–**E**) Three independent experiments, 3 mice/group, 3 LNs/mouse, 3 sections/LN, and 3-5 fields/section. Student’s unpaired, 2-tailed *t* test for 2-group comparisons. Data are represented as mean ± SEM. *P < 0.05; **P < 0.01; ***P < 0.001; ****P < 0.0001. *P* < 0.05 was considered significant.

Since our prior studies demonstrated an immunomodulatory role for Lama5 in regulating LN niches, we next sought to define the roles of LN FRC–derived Lama4 in homeostasis and immune responses by creating FRC-Lama4 conditional KO *Pdgfrb-Cre^+/–^ × Lama4^fl/fl^* mice. Lama4-floxed and Pdgfrb-Cre DNA sequences were confirmed by genotyping (Supplemental Figure 2, A–C). The *Pdgfrb-Cre^–/–^ × Lama4^fl/fl^* littermates were used as WT controls. In nonhematopoietic LNSCs, the Lama4 transcript was depleted in FRCs of FRC-Lama4–KO mice, but not in BECs or LECs (Figure 2A). This was consistent with single-cell RNA-sequencing (scRNA-Seq) data showing that the promoter gene *Pdgfrb* was exclusively expressed in FRCs, but not in BECs and LECs (Supplemental Figure 2D). Depletion of Lama4 did not affect Lama5 expression in LNSCs (Figure 2A). Immunofluorescence microscopy showed Lama4, but not Lama5, protein was depleted in CR and around HEVs, yielding a significant decrease in the Lama4/Lama5 ratio (Figure 2B). Lama4 and Lama5 protein expression in CD4^+^ and CD8^+^ T cells, B cells, and DCs was not affected by depleting FRC-Lama4 (Supplemental Figure 3). FRC-Lama4–KO and WT mice had similar total numbers and abundance of CD4^+^ and CD8^+^ T cells, B cells, CD11c^+^ conventional DCs (cDCs), and total cells in peripheral LNs (pLNs) and mesenteric LNs (mLNs) (Figure 2C). We detected very few CD44^hi^ and CD44^hi^CD62^–^ cells out of CD4^+^ T and CD8^+^ T cells in naive WT and FRC-Lama4–KO LNs (Supplemental Figure 4A), and negligible amounts of anti-dsDNA without differences between naive WT and FRC-Lama4–KO aged mice (Supplemental Figure 4B). Moreover, CD4^+^ T effector cells, including T-bet^+^Th1, GATA3^+^Th2, and RORγt^+^Th17, and CD8^+^ T effector cells, including T-bet^+^Tc1, GATA3^+^Tc2, and RORγt^+^Tc17, were not detected in naive WT or FRC-Lama4–KO mice (Supplemental Figure 5). These results indicated that depletion of FRC-Lama4 did not cause overt immune activation or autoimmunity in the naive state or during aging. In contrast, there was a pronounced loss of PDCA1^+^ plasmacytoid DCs (pDCs) in FRC-Lama4–KO LNs compared with WT (Figure 2C), in particular within the CR, B cell follicles, medulla, and around HEVs. Likewise, cDCs were also decreased in these regions except around HEVs (Figure 3A). Notably, pDCs contribute to tolerance by promoting regulatory Foxp3^+^ Treg differentiation in LNs (21). Hence, we next assessed Tregs in FRC-Lama4–KO LNs.

Flow cytometry demonstrated no difference in thymic Treg (tTreg) (Foxp3^+^Helios^+^) or induced Treg (iTreg) (Foxp3^+^Helios^–^) numbers or content between WT and FRC-Lama4–KO LNs (Supplemental Figure 6). In contrast, we observed decreased Foxp3^+^ Treg intensity in FRC-Lama4–KO LNs by immunofluorescent microscopy, particularly in the T-zone, CR, and around HEVs (Figure 3, B and C). We next analyzed the innate lymphoid cells (ILCs) in LNs (22). By producing diverse cytokines similar to T helper cells, ILCs serve as innate counterparts of T cells and play critical roles in lymphoid organogenesis, tissue remodeling, and antimicrobial immunity (23). We found inflammatory ILC1 increased, but the protective ILC2 and ILC3 subsets decreased in FRC-Lama4–KO LNs (Figure 3, D and E). These results together demonstrated that, as an important ECM component, FRC-Lama4 maintains homeostatic DCs, Tregs, and ILCs in LNs.

### FRC-Lama4 depletion affects FRC chemokine and cytokine secretome.

**Figure 4:**
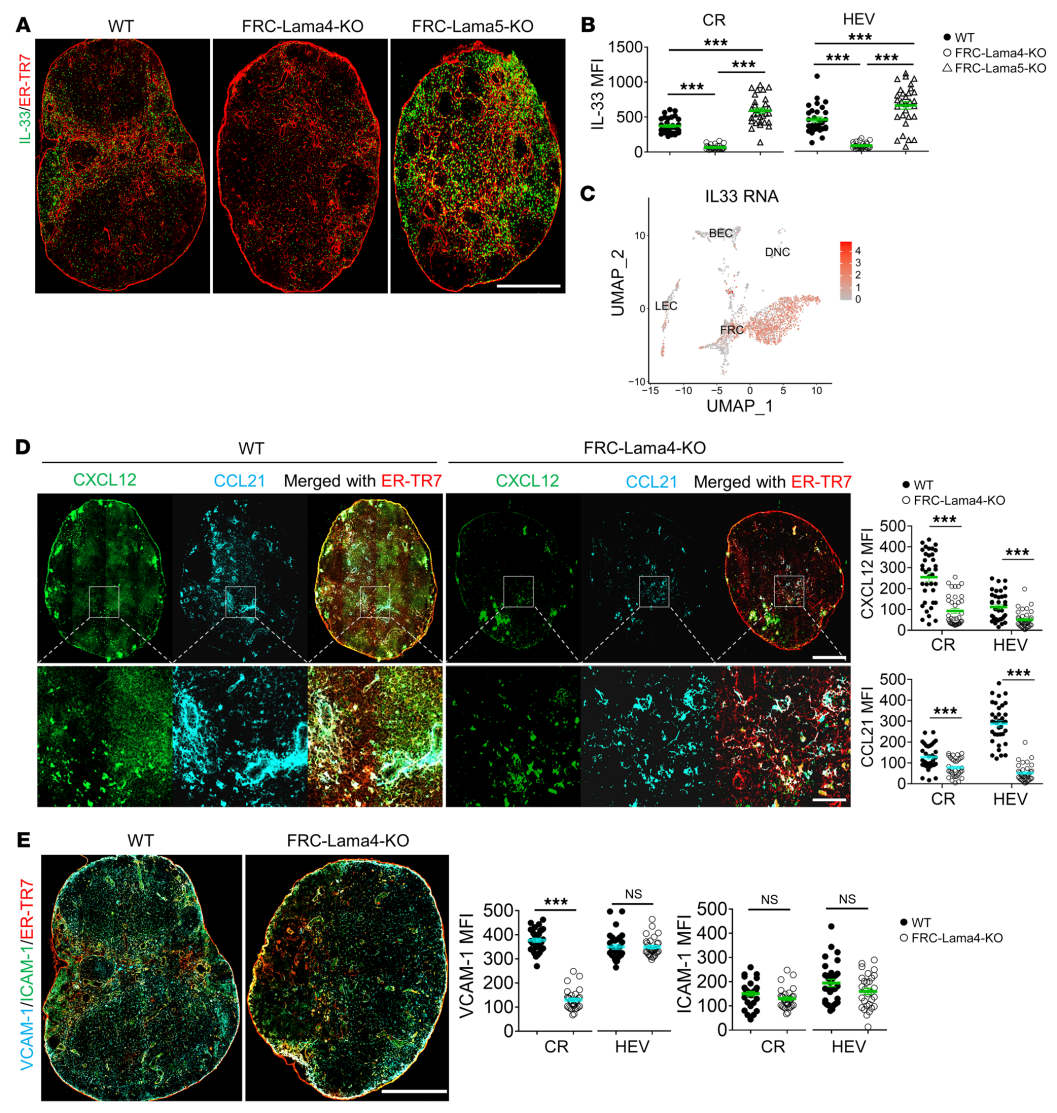
FRC-Lama4 depletion impairs chemokine and cytokine production. (**A**) Whole-mount scanning of FRC-Lama4–KO, FRC-Lama5-KO, and WT mouse LN cryosections stained for IL-33 and ER-TR7. Original magnification, ×20. Scale bar: 500 μm. (**B**) Quantification of IL-33 intensity in LN CR and around HEVs. (**C**) IL-33 gene expression in LNSCs measured by scRNA-seq. (**D**) Whole-mount scanning of FRC-Lama4–KO and WT LN cryosections stained for CXCL12 and CCL21. Original magnification, ×20. Scale bars: 500 μm (upper); 100 μm (lower). Quantification of CXCL12 and CCL21 in LN CR and around HEVs. (**E**) Whole-mount scanning of FRC-Lama4–KO and WT LN cryosections stained for VCAM-1 and ICAM-1. Original magnification, ×20. Scale bar: 500 μm. Quantification of VCAM-1 and ICAM-1 in CR and around HEV. Representative of 3 independent experiments with 3 mice/group, 3 LNs/mouse, 3 sections/LN, and 3 to 5 fields/section. One-way ANOVA with Tukey’s multiple comparisons test for multiple group comparison (**B**). Student’s unpaired, 2-tailed *t* test for 2-group comparisons (**D** and **E**). Data are represented as mean ± SEM. ***P < 0.001. *P* < 0.05 was considered significant.

The alteration in FRC-Lama4–KO LN cellularity could be due to cell differentiation, proliferation, and/or recruitment (e.g., ingress, egress, or retention) in LNs. With these possible mechanisms in mind, we compared the cell growth factors, chemokines, cell adhesion molecules, and vasculature between FRC-Lama4–KO and WT LNs. Immunofluorescent staining of WT LNs revealed that IL-33 was primarily located in FRC-enriched regions, including CR, deep T-zone, and medulla (Figure 4, A and B). This aligned with scRNA-Seq data showing that *Il33* was predominantly expressed in FRCs, but not LECs and BECs (Figure 4C). Relative to WT controls, FRC-Lama4–KO LNs had less IL-33 expression in the CR and around HEVs (Figure 4, A and B). In contrast, FRC-Lama5–KO LNs, in which we previously observed a relative tolerogenic niche (19), had significantly more IL-33 expression. The IL-33–enriched area overlapped with Tregs (Figure 3B and Figure 4A), suggesting that IL-33 contributed to a homeostatic LN niche through supporting Tregs, while depleting FRC-Lama4 impaired this niche. We next screened cytokines and chemokines predominantly produced by FRCs through scRNA-Seq and compared their expression between FRC-Lama4–KO and WT controls using real-time PCR (RT-PCR). Inflammatory chemokines, such as *Cxcl2*, *Cxcl9*, and *Cxcl10*, increased, while the chemokines and cytokines that favor T cell migration and survival, including *Cxcl12*, *Ccl19*, *Ccl21*, *Il7*, *Il15* (9, 24–26), decreased in FRC-Lama4–KO LNs (Supplemental Figure 7). Confirming the transcriptional data, the CCL21 and CXCL12 proteins were particularly decreased in the CR and around HEVs (Figure 4D). The adhesion molecule VCAM-1, which plays important roles in regulating DC migration and T cell retention in LNs (27), was also decreased in FRC-Lama4–KO LNs compared with WT controls, while FRC-Lama4–KO and WT LNs had similar ICAM-1 expression (Figure 4E). Overall, these changes demonstrate FRC-Lama4 contributes to a homeostatic LN niche. Depleting Lama4 altered the LN microenvironment by affecting the FRC secretome, including upregulation of inflammatory cytokines and downregulation of adhesion molecules and immune cell supportive chemokines.

### FRC-Lama4 depletion impairs LN vasculature and conduits.

**Figure 5:**
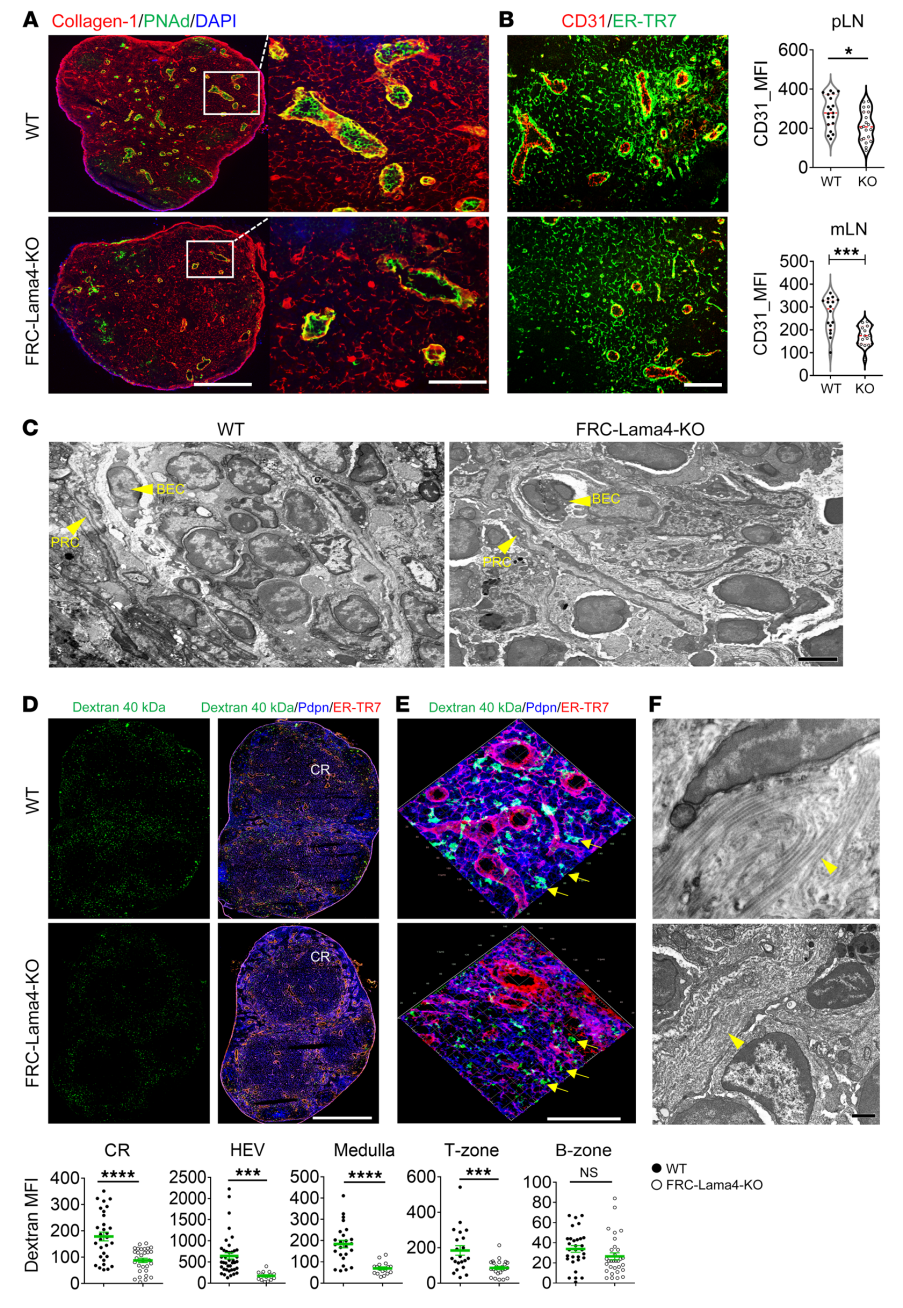
FRC-Lama4 depletion impairs LN vasculature. (**A**) Whole-mount scanning of FRC-Lama4–KO and WT LN cryosections stained for collagen 1, PNAd, and DAPI. Original magnification, ×20. Scale bars: 500 μm (left); 100 μm (right). (**B**) Representative fluorescent images of LN cryosections stained for ER-TR7 and CD31. Scale bar: 100 μm. Quantification of CD31 in HEV areas of pLNs and mLNs. (**C**) TEM images showing HEVs in WT and FRC-Lama4–KO mouse LNs (longitudinal section). Original magnification, ×1100. Scale bar: 2 μm. (**D** and **E**) FRC-Lama4–KO and WT mice injected s.c. with 40 kDa dextran-FITC; draining inguinal LNs harvested 5 minutes later. Whole-mount scanning of LN cryosections stained for ER-TR7 and Pdpn. Original magnification, ×20. Scale bars: 500 μm. Quantification of dextran in various LN regions. (**E**) 3D confocal images of CR; arrows indicate conduits. Original magnification, ×40. Scale bar: 100 μm. (**F**) TEM images of WT and FRC-Lama4–KO mouse LNs (longitudinal section; arrowheads show collagen fibers). Original magnification, ×6500. Scale bar: 500 nm. Representative of 3 independent experiments with 3 mice/group, 3 LNs/mouse, 3 sections/LN, and 3 to 5 fields/section. Student’s unpaired, 2-tailed *t* test for 2-group comparisons. Data are represented as mean ± SEM. *P < 0.05; ***P < 0.001; ****P < 0.0001. *P* < 0.05 was considered significant.

The LN architecture is primarily supported by the fibroblastic reticular network and comprises tightly interconnected CD31^+^ blood vessels, Lyve-1^+^ or Prox-1^+^ lymphatic sinuses, and FRC-ensheathed conduits. LN blood vessels, specialized BECs with cuboidal morphology, selectively recruit blood-borne lymphocytes into the developing and homeostatic LNs via HEVs. Compared with WT controls, the HEVs, identified by PNAd (Figure 5A) or CD31 (Figure 5B), decreased in size and numbers in FRC-Lama4–KO pLNs and mLNs. In contrast, Lyve-1^+^ and Prox1^+^ lymphatic vessels were not affected by depleting FRC-Lama4 (Supplemental Figure 8). The HEVs were further analyzed at higher resolution with transmission electron microscopy (TEM). The HEVs in WT littermates had complete endothelial barriers with well-lined BECs on the luminal side and perivascular reticular cells (PRCs), an FRC subset ensheathing the abluminal side of blood vessels (Figure 5C). In contrast, the HEVs in FRC-Lama4–KO LNs had discontinuous and thinner HEV layers, fewer PRCs and fibers surrounding HEVs, and fewer intraluminal lymphocytes (Figure 5C). These results together indicate that FRC-derived Lama4 is required for development and maintenance of HEV structure.

The conduit system channels small molecules (<70 kDa), such as chemokines, cytokines, and antigens, in lymph from the subcapsular sinus to inner LN compartments (28–31). FRC-derived collagen I and IV assemble fibrillar chains, wrapping the conduits and providing tensile strength (2). Therefore, we visualized the conduit system dLNs by injecting dextran-FITC (40 kDa) s.c. (32). In both WT and FRC-Lama4–KO LNs, the conduit network was widely spread throughout the LN, but enriched in the T-zone, CR, around HEVs, and in medullary regions. Notably, FRC-Lama4–KO LNs had lower dextran intensity than WT (Figure 5D), indicating lower density of conduits. Conduits in FRC-Lama4–KO CRs were smaller and more sparse than in WT, with conduits labeled with dextran, stained for ER-TR7 and Pdpn, and then visualized with 3D reconstruction of confocal images (Figure 5E). Pdpn intensity in FRC-Lama4–KO LNs was lower than in WT, particularly in the CR, HEV, T-zone, and medullary regions (Supplemental Figure 9), indicating a reduced reticular network. The conduits have a central core of collagen fibers with the abluminal basement membrane wrapped with FRCs (29, 33, 34). Under TEM visualization, the FRC-Lama4–KO LNs had fewer and more disorganized collagen fibers compared with WT (Figure 5F), suggesting a defective conduit system. Taken together, these results indicate that FRCs, and Lama4 in particular, play important roles in maintaining LN conduit numbers, density, size, and structure.

### FRC-Lama4 is required for lymphocyte migration into LNs.

**Figure 6:**
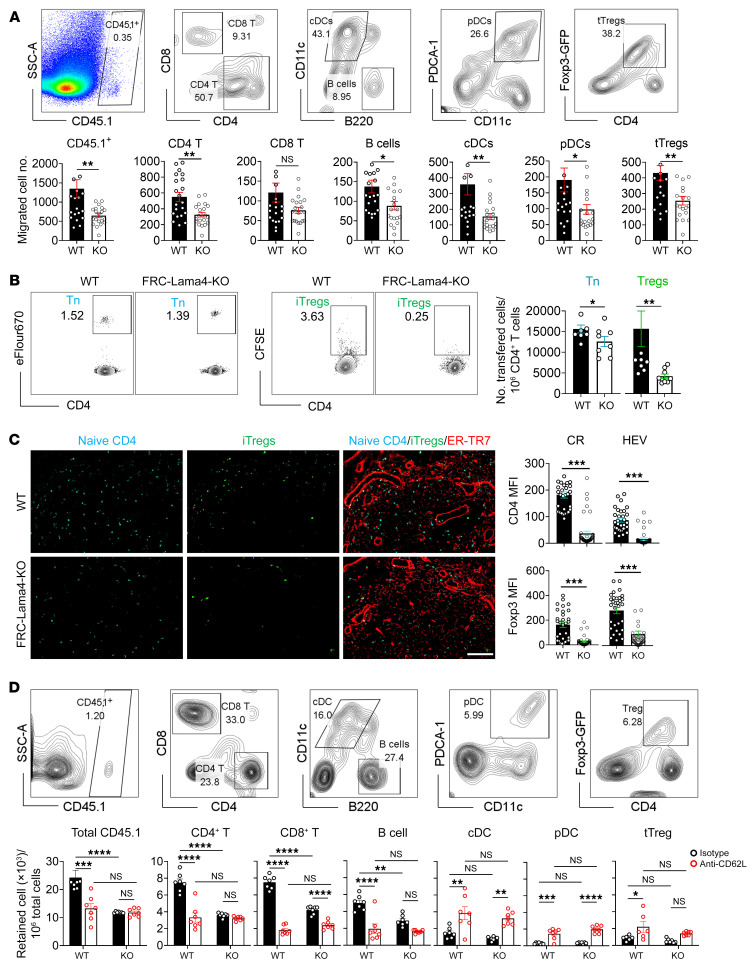
FRC-Lama4 depletion affects lymphocyte entry into LNs. (**A**) 10^7^ CD45.1^+^ splenocytes transferred i.v. into CD45.2^+^ WT and FRC-Lama4–KO recipients. After 1 hour, LNs were harvested and total migrated CD45.1^+^ cells, and CD45.1^+^ CD4^+^ T, CD8^+^ T cells, B cells, cDCs, pDCs, and Foxp3-GFP^+^ tTregs were counted in each LN. Gating strategy (upper) and data summary (lower) of migrated cells. (**B** and **C**) 2 × 10^6^ CFSE^+^ iTregs and 2 × 10^6^ eFlour 670^+^ CD4^+^ T cells transferred i.v. to FRC-Lama4–KO and WT mice. After 16 hours, LNs were harvested and transferred cells measured. (**B**) Flow cytometry gating strategy (left, values show percentage); number of transferred naive CD4^+^ T cells and iTregs relative to 10^6^ total CD4^+^ T cells in LNs. (**C**) LN cryosections for CD4^+^ and iTregs and ER-TR7. Original magnification, ×20. Scale bar: 100 μm. Quantification of naive CD4^+^ T cells and iTregs in CR and HEV. (**D**) 10^7^ CD45.1^+^ splenocytes transferred i.v. into CD45.2^+^ WT and FRC-Lama4–KO recipients. Eighteen hours later, recipients received 100 μg anti-CD62L mAb or isotype i.v. After an additional 18 hours, transferred cells in LNs were analyzed. Gating strategy (upper) and data summary (lower) of migrated cell frequency in recipient LNs. (**A**–**D**) Values in gating strategy show percentages. Representative of 3 independent experiments with 3 mice/group, 5 LNs/mouse, 3 sections/LN, and 3 to 5 fields/section. Student’s unpaired 2-tailed *t* test for 2-group comparisons. Two-way ANOVA with multiple comparisons test. Data are represented as mean ± SEM. *P < 0.05; **P < 0.01; ***P < 0.001; ****P < 0.0001. *P* < 0.05 was considered significant.

The chemokines, HEVs, and conduit networks analyzed above are critical infrastructure allowing blood-borne immune cells to enter LNs. We hypothesized that changes in these would affect lymphocyte homing to and retention in LNs and would subsequently affect transplant tolerance. To test this, we examined the homing of various leukocyte subsets by transferring CD45.1^+^ splenocytes i.v. into CD45.2^+^ WT and FRC-Lama4–KO recipients. One hour after transfer, sufficient for blood migration (35), fewer total CD45.1^+^ cells and CD45.1^+^ CD4^+^ T cells, CD8^+^ T cells, B cells, cDCs, pDCs, and Foxp3-GFP^+^ Tregs were present in KO LNs compared with WT (Figure 6A). These results demonstrated that depletion of Lama4 impaired homing of various leukocytes. To assess cell accumulation in LNs over time, eFluor670-labeled naive CD4^+^ T cells and Foxp3-GFP^+^ Tregs were transferred into WT and KO mice, and LN occupancy was measured 16 hours later. Compared with the WT, KO LNs had fewer CD4^+^ T cells and Tregs (Figure 6B). Histological analysis revealed fewer CD4^+^ T cells and Tregs in the CR and around HEVs of KO LNs (Figure 6C). Hence, depleting FRC-Lama4 also blunted T cell retention in LNs during a time frame that would permit several rounds of entry and egress from LNs. Having revealed the effects of Lama4 on entry and accumulation above, we next determined whether FRC-Lama4 depletion affected LN residency and egress. Eighteen hours after transferring CD45.1^+^ splenocytes to CD45.2^+^ WT and FRC-Lama4–KO recipients,these mice were then treated with anti-CD62L mAbs to prevent further immune cell entry to LNs via HEVs. After an additional 18 hours, transferred cells in LNs were measured. Blocking CD62L in WT demonstrated that B cells and CD4^+^ and CD8^+^ T cells efficiently exited the LNs, but cDCs, pDCs, and Tregs did not, as evidenced by their relative increase (Figure 6D). This indicated that DCs and Tregs were more sessile in WT LNs. In FRC-Lama4–KO LNs, blocking cell entrance with CD62L mAbs did not result in further changes in transferred cell numbers compared with WT, showing that those cells that entered the FRC-Lama4–KO LNs had normal characteristics of retention and egress (Figure 6D). Overall, these results demonstrated that impaired cell entrance but not egress contributed to the reduced cell numbers in FRC-Lama4–KO LNs. Collectively, these results indicated that FRC-Lama4 is necessary for lymphocyte recirculation through LNs. Given the alterations in Tregs and DCs both at homeostasis (Figures 2 and 3) and after transfer (Figure 6) and the critical roles they play in adaptive immune responses and tolerance (36), we next studied the effects of FRC-Lama4 on alloimmunity.

### Ablation of FRC-Lama4 promotes T cell alloimmunity.

**Figure 7:**
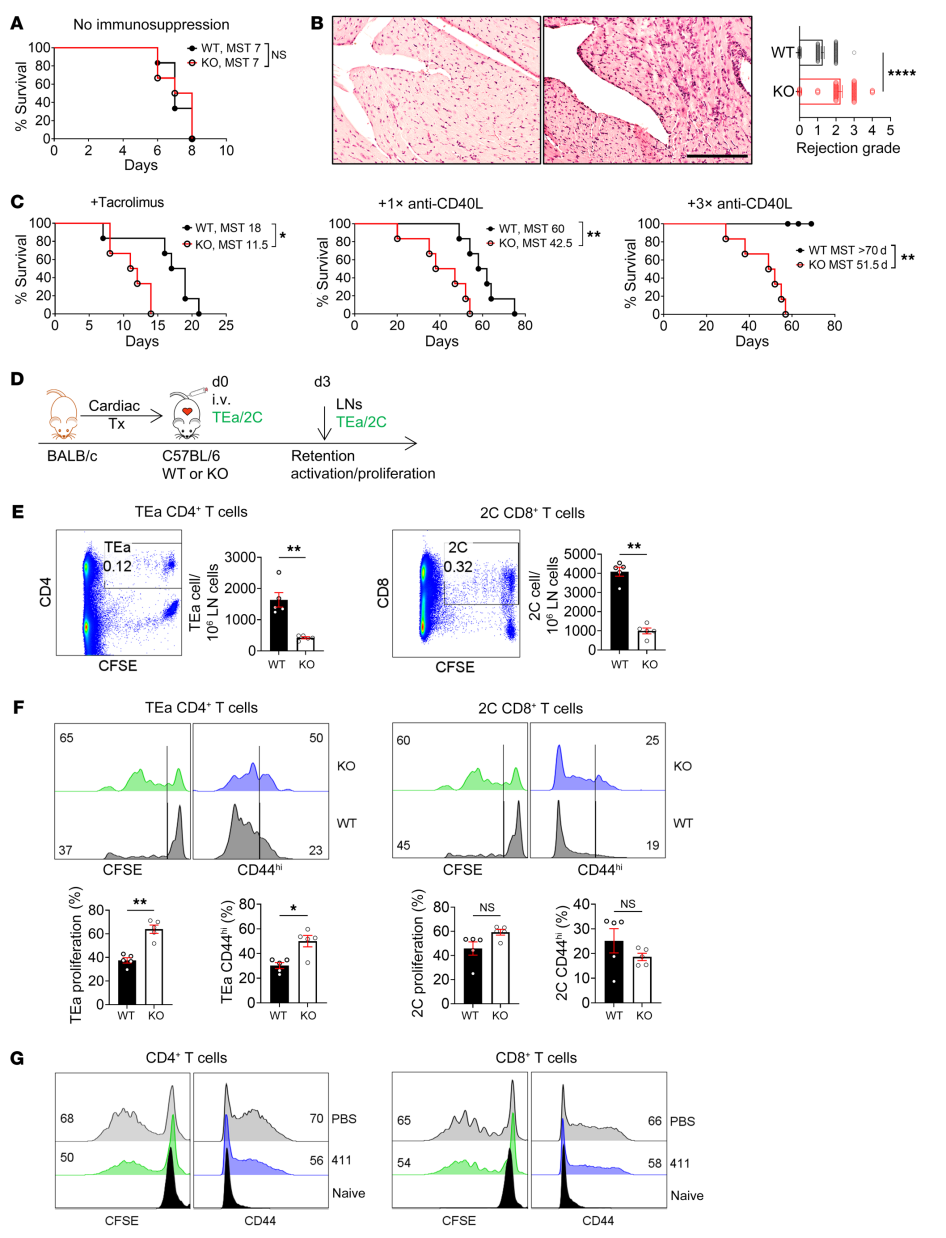
Ablation of FRC-Lama4 promotes T cell alloimmunity. (**A**–**C**) C57BL/6 WT and FRC-Lama4–KO received BALB/c cardiac allografts. Allograft survival (**A**) without immune suppression or (**C**) with tacrolimus (2 mg/kg/day s.c.), 1 dose (1×, 250 μg, i.v. day 0), or 3 doses (3×, 250 μg/dose, i.v. days 0, 4, 7) of anti-CD40L mAb; log-rank (Mantel-Cox) test for graft survival, median survival time (MST), 6 mice/group. (**B**) H&E staining of heart grafts in WT (left) and FRC-Lama4-KO recipient mice (right) 3 days after transplantation, and rejection grade. Scale bar: 100 μm. (**D**–**F**) (**D**) Schematic of cardiac transplantation with adoptive transfer of alloantigen-specific TEa CD4^+^ and 2C CD8^+^ cells. CFSE-stained TEa plus 2C cells (each 2 × 10^6^) injected i. v. into WT and FRC-Lama4–KO recipients on day 0. Recipient LNs harvested on day 3 to assess TEa and 2C cell numbers and responses. (**E**) Number, (**F**) proliferation, and CD44 expression at day 3. (**G**) CFSE^+^, CD4^+^, and CD8^+^ T cells cocultured with coated laminin 411 (2 μg/mL) or PBS and activated by coated CD3 mAb (5 μg/mL) and soluble CD28 mAb (1 μg/mL). CFSE and CD44 analyzed 3 days after activation. (**E**–**G**) Values show percentage. Data representative of 3 independent experiments; 3 mice/group. (**B**, **E**, and **F**) Student’s unpaired 2-tailed t test for 2-group comparisons. Data are represented as mean ± SEM. *P < 0.05; **P < 0.01; ****P < 0.0001. *P* < 0.05 was considered significant.

We next examined the influence of FRC-Lama4 on allogeneic T cell responses in murine cardiac transplantation. Without immunosuppression, allografts in FRC-Lama4–KO and WT recipients were both rejected by 8 days after transplant (Figure 7A), but the grafts in FRC-Lama4–KO recipients had greater lymphocytic infiltration by 3 days after transplantation (Figure 7B). Low-dose tacrolimus–treated FRC-Lama4–KO recipients had significantly shorter allograft median survival time (MST) (11.5 days versus 18 days in WT; Figure 7C). When mice received a single dose of anti-CD40L mAbs, FRC-Lama4–KO recipients had significantly decreased allograft survival compared with WT (MST, 42.5 versus 60 days; Figure 7C). A tolerogenic regimen of 3 doses of anti-CD40L (250 μg/dose, days 0, 4, 7 after transplantation) induced prolonged survival in WT, but not in FRC-Lama4–KO recipients (Figure 7C). Three weeks after transplantation, alloantibodies in blood serum were not detected in WT recipients, which did not show a difference compared with the naive mice. In sharp contrast, the FRC-Lama4–KO recipients produced dramatically more IgG and IgM alloantibody than WT (Supplemental Figure 10). These results demonstrated that absence of Lama4 in FRCs prevented tolerance induction. To elaborate regarding the molecular changes caused by the alloantigens from BALB/c graft, we adoptively transferred T cell receptor transgenic (TCR Tg) CD4^+^ T cells from TEa mice and CD8^+^ T cells from 2C mice into the recipients immediately after transplantation. The TEa and 2C T cells can recognize BALB/c donor-specific antigens (Figure 7D). Three days later, we observed that fewer TEa and 2C T cells were retained in the FRC-Lama4–KO recipient LNs (Figure 7E), but these cells displayed greater proliferation compared with what occurred in WT (Figure 7F). TEa CD4^+^ T cells, but not 2C CD8^+^ T cells, in FRC-Lama4–KO LNs had higher expression of CD44 (Figure 7F), indicating greater activation. We confirmed the effects of Lama4 on T cells by coculturing laminin α4β1γ1 (laminin 411) trimer with CD4^+^ and CD8^+^ T cells activated by CD3 and CD28 mAbs for 3 days. Commensurate with the in vivo results in transplantation, laminin 411 trimer suppressed CD4^+^ and CD8^+^ T cell proliferation and activation (Figure 7G). Overall, these results indicate that antigen-specific T cells were more activated in the LN niches lacking FRC-Lama4.

### FRC-Lama4 regulates Treg versus T effector balance in cardiac transplants.

**Figure 8:**
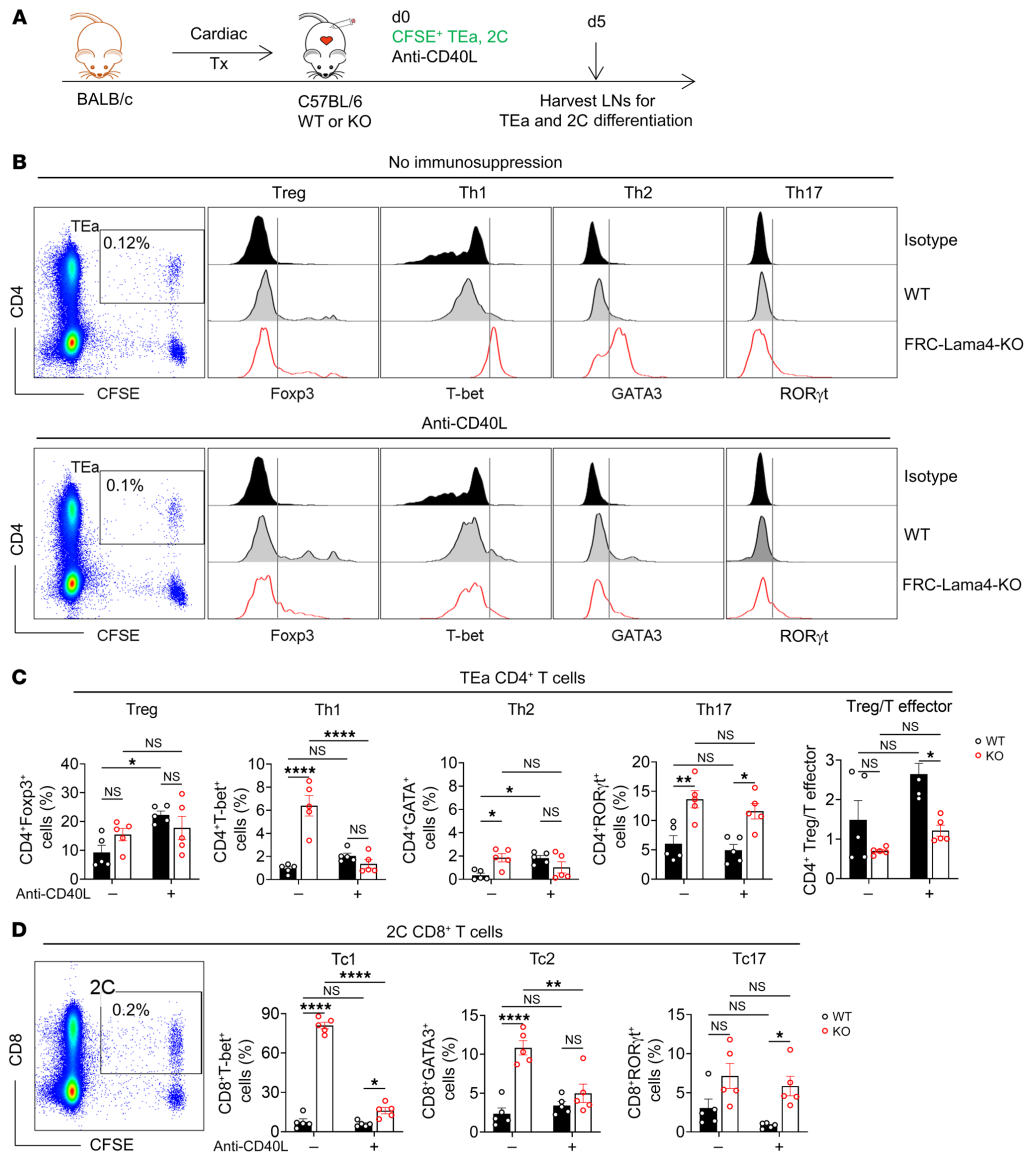
FRC-Lama4 regulates Treg versus T effector balance in cardiac transplants. (**A**) Schematic of cardiac transplantation with immunosuppression and transfer of alloantigen-specific, TCR Tg TEa CD4^+^ T cells and 2C CD8^+^ T cells. On day 0, CFSE-stained TEa plus 2C cells (2 × 10^6^ each) with or without 250 μg anti-CD40L injected i.v. to WT or FRC-Lama4–KO recipients. LNs harvested on day 5 to assess TEa and 2C cell differentiation. (**B**) Representative gating of TEa CD4^+^ T cells and differentiation to Foxp3^+^Treg, T-bet^+^Th1, GATA3^+^Th2, and RORγt^+^Th17 cells from Foxp3^–^ TEa cells. (**C**) Data summary of TEa differentiation and ratio of Treg/T effector cells (**D**) Gating of 2C cells and data summary of 2C differentiation (gating of T-bet^+^Tc1, GATA3^+^Tc2, and RORγt^+^Tc17 cells same as TEa effector cells in **B**). Data are representative of 3 independent experiments; 3 mice/group. Two-way ANOVA with multiple comparisons test for multiple comparisons of each group. Data are represented as mean ± SEM. *P < 0.05; **P < 0.01; ****P < 0.0001. *P* < 0.05 was considered significant.

To further assess the effects of FRC-Lama4, we analyzed the differentiation of TEa and 2C cells to Tregs and T effector cells with or without anti-CD40L mAb immunosuppression (Figure 8, A and B). Without anti-CD40L, depleting FRC-Lama4 significantly enhanced TEa CD4^+^ T cell differentiation into effector Th1, Th2, and Th17 cells, but not Tregs (Figure 8C). A single dose of anti-CD40L mAbs promoted TEa CD4^+^ T cell differentiation to Tregs in WT but not FRC-Lama4–KO LNs (Figure 8C). Anti-CD40L mAbs also prevented increased Th1 and Th2 differentiation in the KO, but not Th17. With or without anti-CD40L mAbs, FRC-Lama4–KO had lower CD4^+^ Treg/T effector ratios. For 2C CD8^+^ T cells, without immunosuppression, differentiation into effector Tc1 and Tc2 was greater in FRC-Lama4–KO (Figure 8D). There was also a slight, but not statistically significant, increase in Tc17 in FRC-Lama4–KO. Anti-CD40L mAbs prevented the increased Tc1 and Tc2 differentiation in the FRC-Lama4–KO, but was ineffective in decreasing Tc17 differentiation. These results demonstrate that FRC-Lama4 regulates immunity and suppression through T cell differentiation to Tregs relative to Th and Tc effectors.

### FRC-Lama4 regulates alloimmunity in lung transplantation.

**Figure 9:**
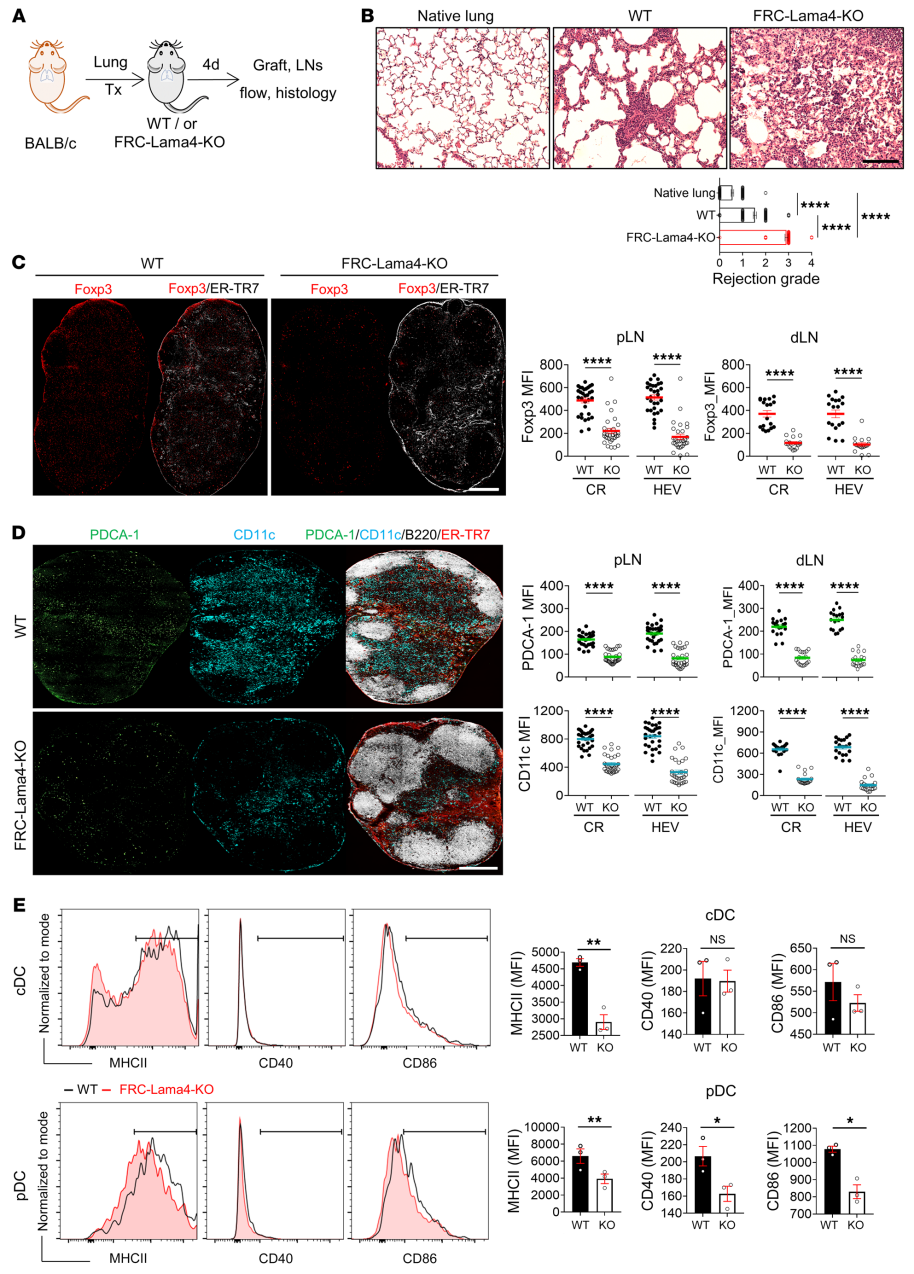
FRC-Lama4 regulates alloreactivity in lung transplants. (**A**) Schematic of lung transplantation. BALB/c donors, C57BL/6 WT, or FRC-Lama4–KO recipients. Grafts and LNs harvested 4 days after transplantation. (**B**) H&E staining of native lung from BALB/c mice, lung grafts in WT and FRC-Lama4–KO recipients. Scale bar: 100 μm. Evaluation of rejection grade. (**C**) Whole-mount scanning of recipient pLN cryosections stained for Foxp3 and ER-TR7. Original magnification, ×20. Scale bar: 500 μm. Quantification of Foxp3 intensity in CR and around HEVs in recipient pLNs and lung dLNs. (**D**) Whole-mount scanning of recipient pLN cryosections stained for PDCA-1, CD11c, ER-TR7, and B220. Original magnification, ×20. Scale bar: 500 μm. Quantification of PDCA-1 and CD11c intensity in CR and around HEVs in recipient pLNs and lung dLNs. (**E**) Gating and summary of MHCII, CD40, and CD86 on cDCs and pDCs in WT and FRC-Lama4–KO recipient pLNs. (**E**) Representative of 2 independent experiments with 3 mice/group, 3 LNs/mouse, 3 sections/LN, 3 to 5 fields/section. Student’s unpaired, 2-tailed *t* test for 2-group comparisons. Data are represented as mean ± SEM. *P < 0.05; **P < 0.01; ****P < 0.0001. *P* < 0.05 was considered significant.

We examined the role FRC-Lama4 played in another model of vascularized orthotopic murine lung allografting. Lungs from BALB/c donors were transplanted into C57BL/6 FRC-Lama4–KO and WT recipients (Figure 9A). Four days after transplantation, lung allografts in FRC-Lama4–KO recipients had significantly greater lymphocyte infiltration and increased rejection score compared with WT (Figure 9B). WT and FRC-Lama4–KO recipient LNs had the same frequency, proliferation, and activation of total B and T lymphocytes (Supplemental Figure 11, A–C). However, there were significantly fewer Foxp3^+^ Tregs distributed in the CR and around HEVs in FRC-Lama4–KO recipient pLNs and lung dLNs (Figure 9C). cDC and pDC frequencies were lower in FRC-Lama4–KO recipient LNs (Supplemental Figure 11, A–C), and pDCs and cDCs were clearly reduced in the CR and around HEVs in both pLNs and lung dLNs (Figure 9D). DCs in the FRC-Lama4–KO LNs had equivalent Ki67 expression (Supplemental Figure 11C) and some small reductions in expression of MHC II, CD40, or CD86 compared with WT (Figure 9E), suggesting that alterations in LN structure and chemokines resulted in less migration or retention of activated DCs in FRC-Lama4–KO LNs. Overall, these results demonstrate that depleting FRC-Lama4 results in more intense lymphocyte infiltration into allografts associated with fewer Tregs and DCs in lung dLNs and pLNs.

In summary, FRC-Lama4 contributes to a tolerogenic LN niche through retention of T cells, constraining T cell immunity, and directing T cell differentiation toward suppressive phenotypes. Ablation of FRC-Lama4 impairs the LN microenvironment and hence undermines suppression and tolerance.

## Discussion

The LN vascular–stromal compartments shape immune responses by presenting antigens, recruiting DCs and lymphocytes, and regulating immune cell destinations (e.g., migration, survival, and differentiation) (37). The FRCs, mostly Pdpn^+^Pdgfrα^+^Pdgfrβ^+^SMA^+^, mark the most critical stromal cells that support LN architecture, encase vessels, and provide various functional ECM components to regulate immune cells (38). Laminins belong to ECM and regulate immune cell migration (17, 39) and functions (18). The present study revealed the regulatory roles of LN FRC–derived Lama4 on LN niches under homeostatic conditions and during alloimmune stimulation. Using the CCL19/iDTR and the FRC-Lama4 conditional KO mouse strains, we revealed that Lama4 was responsible for the regulatory effects of FRCs on homeostatic LN architecture and cellularity and chemokine/cytokine productions. Depletion of Lama4 interfered with HEV and conduit system development, altered the FRC proteome secretion landscape, and resulted in fewer LN Tregs, cDCs, pDCs, and ILC2s. Functionally, in vivo migration or LN retention of Tregs, pDCs, cDCs, B cells, and CD4^+^ and CD8^+^ T cells was reduced in the FRC-Lama4–KO LNs. In lung and cardiac allograft transplantation, FRC-Lama4–KO recipients displayed more severe histological rejection with reduced allograft survival. Mechanistically, Lama4 promotes the migration but suppresses the proliferation and activation of CD4^+^ and CD8^+^ T cells. Ablation of FRC-Lama4 resulted in altered T cell differentiation into T effector cells. Immunosuppression was not efficient in recipients lacking FRC-Lama4, as evidenced by less Treg induction and shorter allograft survival. Notably, depleting FRC-Lama4–KO resulted in expanded B cell follicles at homeostasis (Figure 3B) and after lung transplantation (Figure 9D). Overall, the present study demonstrates that Lama4 is a critical FRC-derived ECM component, essential for a tolerogenic LN microenvironment and thus an efficient immunomodulatory target.

Depletion of Lama4 led to decreased Pdpn expression on FRCs (Supplemental Figure 9), indicating that Lama4 is indispensable for maintaining the FRC phenotype. Relative to WT, the Lama4-KO FRCs upregulated expression of inflammatory chemokines CXCL9 and CXCL10, but downregulated T cell–supportive CCL19, CXCL12, CCL21, IL-6, IL-7, and IL-15 (Supplemental Figure 7). CCL21, CXCL12, IL-33, and VCAM-1, which bridge stromal-immune cell crosstalk (40), were decreased in the CR and around HEVs in Lama 4 KO LNs (Figure 4). IL-33 is an IL-1 family cytokine produced by FRCs and endothelial and neuronal cells (41–43). Our scRNA-Seq data and fluorescent staining indicate that IL-33 is predominantly produced by FRCs relative to LECs and BECs (Figure 4). IL-33 supports protolerant Tregs, ILC2s, and macrophages, which play crucial roles in restricting inflammation (44–46). Therefore, we speculate that downregulated IL-33 in FRC-Lama4–KO LNs contributed to the decreased ILC2 and Tregs and that decreased CCL21 and CXCL12 also caused decreased LN homing of Tregs and other lymphocytes.

HEVs are composed of endothelial cells and perivascular sheathing FRCs. They effectively recruit blood-borne lymphocytes and DCs that migrate between tissues and LNs to differentiate into functional subsets or deliver antigens, thereby generating immune responses (47). FRC-Lama4 ablation decreased HEV numbers and impaired their expression of CD31 and PNAd (Figure 5). Subsequently, the homing of CD4^+^ and CD8^+^ T cells, B cells, and DCs was blunted in FRC-Lama4–KO (Figure 6). In HEV luminal migration, CCL21, PNAd, and ICAM-1 immobilized on the HEV luminal side regulate rolling, activation, and arrest of T cells, respectively (48). Blocking PNAd suppresses T and B lymphocyte postluminal migration and parenchymal distribution (47), indicating that PNAd is indispensable for the transmigration of lymphocytes across HEVs. FRCs also produce various ECM components for building the conduit system. The CCL19^hi^ TRC subset secretes collagen 14 and fibromodulin to build the conduit system (5). Consistent with this, the FRC-Lama4–KO mouse LNs had defective conduits, as evidenced by fewer tracer signals, disorganized morphology, and impaired collagen fiber organization under fluorescent microscopy and TEM (Figure 5). Overall, the results demonstrated that FRC-Lama4 is indispensable for the development and function of HEVs and the conduit system.

Shortly after allograft transplantation, FRC-Lama4 depletion promoted TEa CD4^+^ T cell differentiation into effector Th1, Th2, and Th17 cells and enhanced 2C CD8^+^ T cell differentiation into effector Tc1, Tc2, and Tc17 cells in recipient LNs. This is commensurate with our reported in vitro analyses showing that the protein laminin 411 restricts CD4^+^ effector cell differentiation, including that of Th1, Th2, and Th17 cells (18). With and without immunosuppression, FRC-Lama4 depletion resulted in lower CD4^+^ Treg/T effector ratios, indicating a relatively proimmune microenvironment. pDCs also contribute to a suppressive microenvironment by promoting CD4^+^ and CD8^+^ regulatory cell differentiation (21, 49). Hence, we speculate that the decreased pDCs at homeostasis (Figures 2 and 3), after transfer (Figure 6), and after allograft transplantation (Figure 9) in FRC-Lama4–KO may further contribute to the proimmune LN niche. The influence of Lama4 on T cells raises the question as to what molecule(s) transduced the signal from Lama4. To this end, we have tested integrin α_6_ and α-dystroglycan, which are known Lama5 receptors on T cells (19, 50), and other candidates including integrins β_1_, α_3_, α_5_, and α_v_. The pan integrin inhibitor MK-0429, an antagonist for α_v_β_1_, α_v_β_3_, α_v_β_5_, α_v_β_6_, α_v_β_8_, and α_5_β_1_ integrins, was also tested. Neither integrin isoform blocking mAbs nor the pan integrin altered the effects of Lama4 on CD4^+^ T cell proliferation and activation (data not shown), indicating that none of these candidate molecules serve as Lama4 receptors on CD4^+^ T cells. More focused discovery initiatives will be required to elucidate the T cell Lama4 receptors, such as a CRISPR-Cas screen or traditional protein crosslinking–immunoprecipitation–mass spectrometry.

Depleting FRC-Lama4 decreased LN pDCs and cDCs (Figure 3A), which both contribute to shaping the LN vascular-stromal compartment in many ways. DCs maintain homeostatic HEVs and promote LN expansion upon immunological challenge by self-producing VEGFs (51) or by stimulating FRCs to release VEGF-A (52, 53). DC-expressed lymphotoxin regulates HEV endothelial cell proliferation through engagement of the endothelial lymphotoxin β-receptor (54). FRCs and DCs mutually support one another during rapid LN expansion driven by infiltrating and dividing lymphocytes. Physical LN elasticity is maintained by the FRC-Pdpn/DC-CLEC-2 signaling cascade (53). Pdpn induces actomyosin contractility in FRCs through the RhoA/C signaling pathway (53). CLEC-2 engagement rapidly uncouples Pdpn from RhoA/C activation, leading to a relaxed actomyosin cytoskeleton and LN expansion (53). The CLEC-2/Pdpn signaling axis also controls conduit matrix composition (30), suggesting that DCs contribute to the FRC-sheathed conduit system. In addition, signal regulatory protein α^+^ cDCs support the proliferation and survival of FRCs in mouse spleen and LNs through the tumor necrosis factor αa signaling pathway (55, 56). In turn, FRCs also sustain DCs, as shown by Kapoor et al., who identified a Grem1^+^ FRC subset that colocalizes with cDCs in paracortical regions and maintains the homeostasis of lymphoid tissue–resident cDCs (57). Collectively, we speculate that depleting Lama4 impairs the FRC phenotype by reducing the chemokines and stromal elements that produce a DC-favorable microenvironment. In turn, the altered DCs further reduced cues needed for the development of HEVs and the conduit system, which affected immune cell recruitment and influenced alloimmune responses. Hence, FRC-Lama4 serves as a critical ECM component for the dedicated LN vascular–stromal compartment.

Tregs are well known for suppressing immune responses, thereby maintaining homeostasis and transplant tolerance. Unfortunately, attempts to augment suppression or reverse immunity through Treg adoptive transfers are of limited utility due to Treg instability, purity, and altered migration patterns, so that i.v. transferred Tregs are dispersed systemically and lack local specificity (58). Given these concerns regarding isolation, timing, and cell fate, we believe transfer of ex vivo–expanded FRCs to improve the LN microenvironment may be a better way of reversing defects caused by FRC-Lama4 absence. FRC transplantation has been shown to have therapeutic efficacy in different mouse models (59–61). Our previous study demonstrated that depleting FRC-Lama5 can create a tolerogenic LN niche with upregulated Tregs that promotes allotolerance (19). Hence, we believe customizing a Treg-favoring LN niche via FRC manipulation may be a relatively better approach for initiating local tolerance. Future studies will seek to reverse the effects caused by Lama4 deficiency by transplanting Lama4-enriched FRCs.

Laminin-expressing fibroblastic cells are major constituents of the local microenvironment in secondary lymphoid organs and tumor sites. The mechanism or mechanisms underlying the regulatory effects of laminins on LN structure and immune outcomes are highly complex. Our initial report demonstrated that Lama4 and Lama5 regulate T cell migration and allograft fate (17). Subsequently, we showed that Lama4 and Lama5 expression dynamically react to diverse immune and tolerance influences, including cancer, gut microbiota, and allotransplantation in mice (62, 63). We showed melanoma dLNs have relatively higher Lama4 than healthy controls (18), suggesting that Lama4 is a target for modulating the tumor microenvironment. In murine transplantation models, we screened antibodies against laminins (17) and against the Lama5 receptors α_6_ integrin and dystroglycan (19) and demonstrated each could influence graft survival in a predictable fashion. The present work elucidated the cellular behaviors and interactions regulated by FRC-Lama4 under homeostasis and after cardiac and lung allotransplantation. The present work also neatly contrasts with our prior studies of FRC-Lama5–KO, so that Lama5 depletion results in a tolerogenic environment, while FRC-Lama4–KO results in an inflammatory and immunogenic environment. This study takes a concrete step toward a more complete network understanding of LN FRC laminins, broadening the horizon of immune-response manipulation through targeting Lama4 and Lama5. Hence, FRC-derived laminins hold great translational importance for the treatment of diverse immune diseases, transplantation, and tumor immunotherapy. Overall, this study revealed that ECM Lama4 underpins FRC-regulated transplant tolerance, identifying a therapeutic target for immunoengineering.

## Methods

### Mice.

CD45.2^+^ C57BL/6 (H-2^b^) and BALB/c (H-2^d^) mice and CD45.1^+^ C57BL/6 mice were purchased from The Jackson Laboratory. To generate the *Pdgfrb-Cre^+/–^ × Lama4^fl/fl^* mice, *Pdgfrb-Cre^+/–^* mice (64) (a gift from Ralf Adams, Max Planck Institute for Molecular Biomedicine, Münster, Germany) were crossed with *Lama4*-floxed mice (Laminin α4*^fl/fl^* mice, generated by the Transgenic Animal Model Core, University of Michigan, Ann Arbor, Michigan, USA), and backcrossed with C57BL/6 for 10 generations. *Pdgfrb-Cre^–/–^ × Lama4^fl/fl^* littermates were used as WT controls. *Pdgfrb-Cre^+/–^ × Lama5^fl/fl^* mice were previously described (19). Both Lama4 and Lama5 conditional KO mice were healthy, fertile, and without abnormal development or growth. TCR Tg mice expressing the TEa TCR (recognizing I-Ed [Eα52-68] antigen in the context of I-A^b^) were acquired from A.Y. Rudensky (Memorial Sloan Kettering Cancer Center, New York, New York, USA). TCR Tg mice expressing the 2C TCR (recognizes the L^d^ class I MHC antigen SIYRYYGL peptide in the context of the H2^b^ MHC class I molecule; ref. 65) were a gift from Thomas Gajewski (Ludwig Center for Cancer Research, University of Chicago, Chicago, Illinois, USA). Experiments were conducted with age- and sex-matched mice aged 8 to 12 weeks. All animals were housed under specific pathogen–free conditions. For temporary depletion of CCL19^+^FRCs, CCL19/iDTR mice received DT (i.p. 100 ng/d × 5 days; ref. 66). Inguinal, brachial, and axillary LNs were collected on day 2 after the 5 injections. Foxp3-GFP mice were provided by A. Rudensky (67).

### Reagents and antibodies.

Human recombinant laminin 411 (catalog LN411) was from BioLamina. Cell proliferation dyes CFSE (C34557) and eFluor 670 (catalog 65-0840), dextran-FITC (40 kDa, catalog #D1845), and Foxp3 Staining Buffer Set (catalog 00-5523-00) were from Thermo Fisher Scientific. Tacrolimus (USP grade, product number 1642802) and BSA (product number A8531) were from Sigma-Aldrich. IL-2 (catalog 51061-MNAE) and TGF-β (catalog 10804-HNAC) were from Sino Biological US. Mouse CCL21 (catalog RND-AF457) was from R&D Systems. DMEM (catalog 10-013-CV) was from Corning. The pan integrin inhibitor MK-0429 was purchased from MedChemExpress. Antibodies used in this study are shown in Supplemental Table 1.

### Cell preparations.

CD4^+^ T cells were isolated from LNs and spleen using EasySep Mouse CD4^+^ T Cell Isolation Kit (catalog 19852; Stem Cell Technologies). For iTregs, CD4^+^ T cells isolated from Foxp3-GFP mice (5 × 10^4^ cells per well) were cultured in pro-Treg medium containing IL-2 (20 ng/mL), coated anti-CD3ε mAbs (1 mg/mL), anti-CD28 (1 mg/mL), and human TGF-β1 (10 ng/mL). The Foxp3-GFP^+^ Tregs were collected by cell sorting. To obtain mouse LNSCs, FRCs, BECs, and LECs were sorted from the CD45^–^ stromal cells, which were harvested through enzymatic digestion of LN, as described (1). The undigested fibrous material was removed by filtration through a 70 μm nylon mesh. For ex vivo expansion of primary FRCs, the cell mixture from LN digestion was cultured in DMEM with 10% FBS and 1% penicillin/streptomycin. Nonadherent cells were removed after 48 hours, and the remaining attached cells were cultured in the same medium for an additional 5 days. Experiments were performed with the fourth passage of ex vivo–expanded LNSCs, which were predominantly CD31^–^Pdpn^+^ FRCs. Before experiments, FRCs were precoated onto the plates and cultured overnight.

### scRNA-Seq.

After enzymatic digestion of LNs harvested from 3 C57BL/6 mice (female, 12 weeks old) as described above, CD45^–^ cells were purified with the CD45^–^ selection beads and were stained with anti-mouse CD45 before sorting for viable CD45^–^ cells. For scRNA-seq, more than 2 × 10^4^ CD45^–^ LNSCs were run on the 10x Chromium Controller (10x Genomics) to partition single cells into nanoliter-scale droplets containing uniquely barcoded beads and processed for sequencing library preparation using the Chromium Single Cell 3′ Reagent Kit (v3 chemistry) (10x Genomics). Generated cDNA libraries were sequenced on a NovaSeq 6000 sequencing system at the Institute for Genome Sciences at the University of Maryland. 4 × 10^3^ Cells per sample were captured on the 10x Chromium chip; 5 to 10 × 10^4^ reads/cell were obtained with a characterization of 2 to 3 × 10^3^ transcripts/cell. Raw data has been deposited in the NCBI’s Gene Expression Omnibus database (GEO GSE202068).

### Anti-dsDNA analysis.

Anti-dsDNA was measured in sera of aged WT and FRC-Lama4–KO mice that were over 1 year old. 100 μL of blood was collected and allowed to clot at room temperature for 15 minutes. The clot was removed by centrifuging at 2000*g* for 20 minutes at 4°C. The sera were diluted 100-fold in PBS for analysis. Anti-dsDNA was measured using the Mouse anti-dsDNA IgG-specific ELISA Kit (catalog MBS2600477, MyBioSource Inc.).

### Cardiac allograft transplantation.

FRC-Lama4–KO or WT littermates were transplanted with heterotopic cardiac allograft from sex-matched donor BALB/c mice, as previously described (68). Recipients were treated with a single dose of anti-CD40L (250 μg i.v.) or received daily tacrolimus (2 mg/kg/d s.c.) starting on the day of transplantation. Graft function was monitored by abdominal palpation daily until rejection. Six mice per group were transplanted. Alloantibodies were measured in sera of recipients 2 weeks after cardiac transplantation, following the protocol described previously (62). Briefly, 10^5^ P815 (H-2^d^) mastocytoma cells (ATCC) were blocked with anti-CD16/32 mAbs (5 μg/mL in 100 μl HBSS/0.5% BSA) for 15 minutes at room temperature. Mouse sera from recipients or naive negative controls were diluted 1:50 in HBSS/0.5% BSA and then incubated with P815 wells for 30 minutes at 4°C. After washing twice with HBSS/0.5% BSA, cells were stained with fluorophore-labeled IgM and IgG antibodies for 30 minutes at 4°C. Cells were analyzed by flow cytometry.

### Flow cytometry.

To prepare single-cell suspensions, mouse LN and spleen were passed through 70 μm Sterile Cell Strainers (catalog 223633548, Thermo Fisher Scientific). Fc receptors were blocked using anti-CD16/32 (catalog 14-0161-86, eBioscience) before staining with antibodies for surface markers. After staining, cells were washed twice with FACS buffer (PBS with 0.5% w/v BSA) and then were fixed with 4% paraformaldehyde (PFA)/PBS solution (catalog J61899, Alfa Aesar), for 5 minutes. For intracellular staining of transcription factors, cells were fixed and permeabilized with Foxp3 Staining Buffer Set following the manufacturer’s protocol. Cells were washed twice before analysis with an LSR Fortessa Cell Analyzer (BD Biosciences). Data were analyzed with FlowJo software, version 10.6 (Tree Star).

### Immunofluorescence microscopy.

Mouse LNs were excised and frozen in OCT (Sakura Finetek) on dry ice, and 6 μm cryosections were fixed with cold acetone/methanol (1:1) solution for 5 minutes. Antibodies were diluted according to the manufacturer’s protocol. After staining with primary antibodies, sections were blocked with 10% serum of the secondary antibody host and incubated with secondary antibodies for 90 minutes. Slides were fixed with 4% PFA/PBS solution for 5 minutes. After washing slides in PBS for 15 minutes, the sections were incubated with 1% glycerol for 5 minutes. ProLong Gold Antifade Mountant (catalog P36930. Thermo Fisher Scientific) was added before putting the cover slides on. Images were acquired with the EVOS FL Auto 2 Imaging System and Nikon Accu-Scope EXC-500, and analyzed with Volocity image analysis software (Quorum Technologies). The percentage of positive area and MFI were quantified based on at least 3 independent experiments with 3 mice per group, 3 LNs per mouse, 3 sections per LN, and 3 to 5 fields per section.

### Visualization of conduit network.

The conduit networks in dLNs were visualized after s.c. injection of the tracer dextran-FITC (40 kDa) (32). Five minutes after injecting 2.5 μg FITC-dextran, the mice were euthanized and the dLNs harvested for immunofluorescence microscopy.

### Histology: TEM and H&E staining.

Electron microscopy was conducted in the electron microscopy core imaging facility at the University of Maryland. LNs were fixed overnight in 2% PFA/1.5% glutaraldehyde (Electron Microscopy Sciences [EMS]) in 0.1M sodium cacodylate at 4°C for 16 hours. LNs were secondarily fixed for 1 hour in 1% osmium tetraoxide/1.5% potassium ferricyanide at 4°C before treatment with 1% tannic acid in water for 45 minutes at room temperature. Samples were dehydrated in sequentially increasing concentrations of ethanol solutions before embedding in EMBed-812 (catalog 14900, EMS). The 70 nm ultrathin resin sections were cut with a diatome 45° diamond knife using an ultramicrotome (UC7; Leica). Sections were collected on formvar-coated slot grids. Images were acquired under a FEI Tecnai T12 Transmission Electron Microscope (TEM). For H&E staining, lung or heart grafts were harvested and fixed for 2 days in 10% buffered formalin (Thermo Fisher Scientific) and then transferred to 70% ethanol. Samples were embedded in paraffin and then H&E stained. Blinded evaluation of rejection grade was conducted based on greater than 100 H&E staining images (20×) for each group assessed by the International Society for Heart and Lung Transplantation (ISHLT) revised grading criteria of 2004 (ISHLT-2004; ref. 69).

### In vivo migration assay.

In vivo blood migration assays were performed to evaluate T cell migration, as previously published (70). Briefly, mice were i.v. injected with 2 × 10^6^ CFSE^+^ naive CD4^+^ T cells and 2 × 10^6^ eFluor 670^+^ iTregs, stained according to the manufacturer’s instructions. To evaluate the migration efficiency of various lymphocytes and DCs, CD45.2^+^ WT or FRC-Lama4–KO mice were injected with 10^7^ CD45.1^+^ whole cells harvested from CD45.1 spleen and LNs. At 16 hours after injection, the mice were euthanized and CD45.1^+^ CD4^+^, CD8^+^, B220, CD11b, CD11c, and Foxp3-GFP^+^ cells were measured through flow cytometry, or immunofluorescence microscopy. To test the retention of these lymphocytes and DCs in LNs, recipients received 100 μg of blocking anti-CD62L mAbs i.v. 18 hours after cell transfer. The cells remaining in LNs were assessed 18 hours after blocking mAb administration.

### Quantitative RT-PCR.

Quantitative RT-PCR (qRT-PCR) was conducted using the SYBR Green PCR Kit (QIAGEN) and the Applied Biosystems 7900HT Fast Real-time PCR System (Life Technologies). RNA isolation, reverse transcription, and PCR were conducted as described previously (71). Primer sequences are summarized in Supplemental Table 2. PCR was started from a 15-minute 95°C denaturation step, followed by 45 cycles of 15 seconds at 94°C, 20 seconds at 56°C, and 20 seconds at 72°C. Normalized values for specific gene expression were calculated as 2-ddCt. RNA samples were run in triplicate; each experimental group consisted of 3 individual samples.

### Statistics.

In vitro experiments were conducted at least 3 times individually with triplicate samples each time. In vivo migration (3 mice/group) was analyzed at least 2 times. Transplantation experiments were performed 3 times. Graphs were generated using GraphPad Prism software (version 8), and data were presented as the mean ± SEM. Statistical analyses were performed using 2-tailed Student’s *t* test for single variable differences and 1-way or 2-way ANOVA test for multiple comparisons. Statistical analysis of graft survival data was assessed by log-rank (Mantel-Cox) test. *P* < 0.05 was considered statistically significant.

### Study approval.

All protocols were approved by the University of Maryland School of Medicine IACUC.

## Author contributions

JSB and LL conceived the study, designed experiments, interpreted the data and wrote the manuscript. LL, MWS, TZ, WP, XL, JZ, ZM, VS, AK, YS, BM, JW, YX, LW, XF, HR, and ML performed experiments. ZM and YG performed lung transplantation in the ASK lab. RA revised the manuscript and supplied constructive suggestions on interpreting results. SJG and AK revised the manuscript with constructive suggestions.

## Supplementary Material

Supplemental data

## Version 1 07/01/2022

Electronic publication
