# Supplementary material for: Lymph node fibroblastic reticular cells preserve a tolerogenic niche in allograft transplantation through laminin α4
Source: J Clin Invest. 2022 Jul 1;132(13):e156994. doi: 10.1172/JCI156994 (PMC9246384; doi:10.1172/JCI156994)
Supplement: Supplemental data [file jci-132-156994-s010.pdf]

Supplementary figures

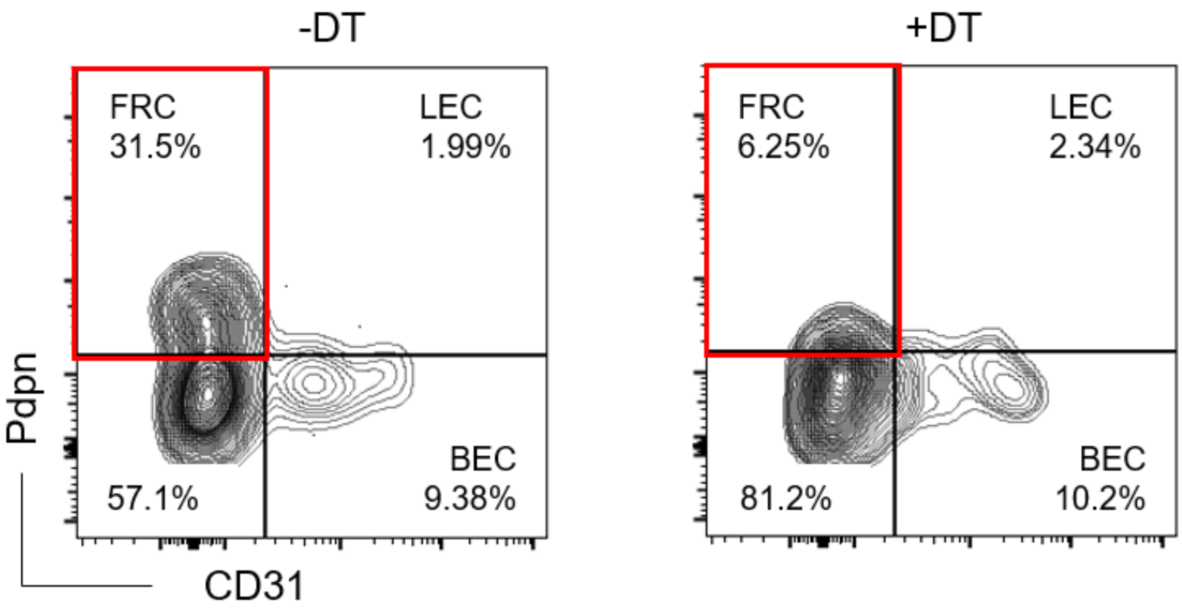

Figure S1. Diphtheria toxin inducible depletion of FRCs in CCL19/iDTR mice. CCL19/iDTR mice received diphtheria toxin (DT) (i.p. 100 ng/day), using PBS for negative control. After 5 days, LNs harvested, and single cell suspension stained for flow cytometry. $Pdpn^+CD31^-$ FRCs, $Pdpn^+CD31^+$ LECs, $Pdpn^-CD31^+$ BECs gated from $CD45^-$ cells.

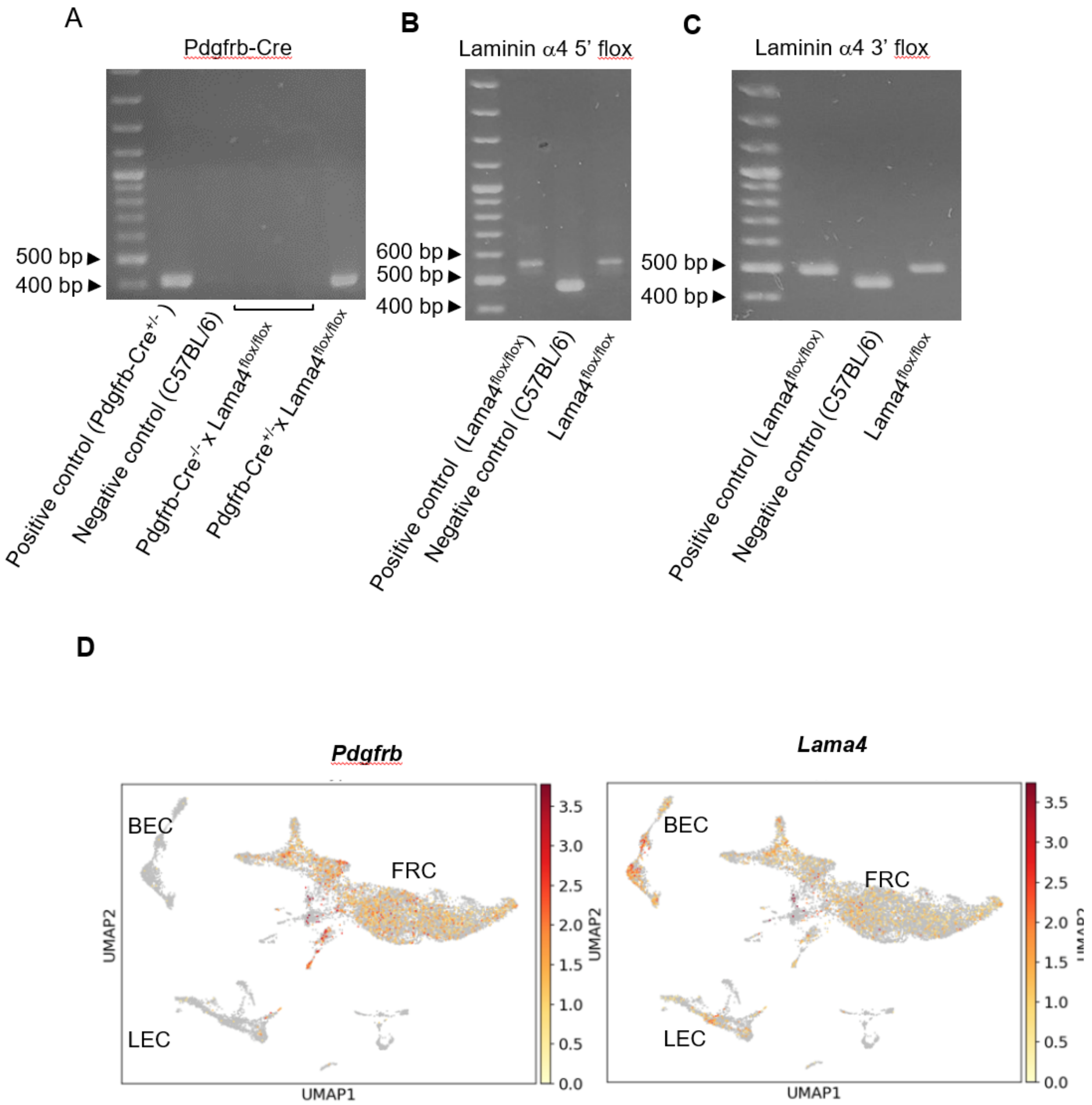

Figure S2. Genotype of Pdgfrb-Cre$^{+/-}$ x Lama4$^{flox/flox}$ mice. Cre inserted in the promotor of Pdgfrb and flox inserted 5' and 3' of Lama4 exon 3; insertions assessed by PCR of gDNA. **A.** Pdgfrb-cre band ~410 bp. Pdgfrb-Cre+/- positive control; C57BL/6 negative control. **B**. 5' flox ~570 bp, WT ~470 bp. Lama4$^{flox/flox}$ positive control; C57BL/6 negative control. **C**. 3' flox ~490 bp, WT ~440 bp. Lama4flox/flox positive control; C57BL/6 negative control. **D**. scRNA-seq analysis showed Lama4 widely expressed in LNSCs, but Pdgfrb exclusively expressed in FRCs.

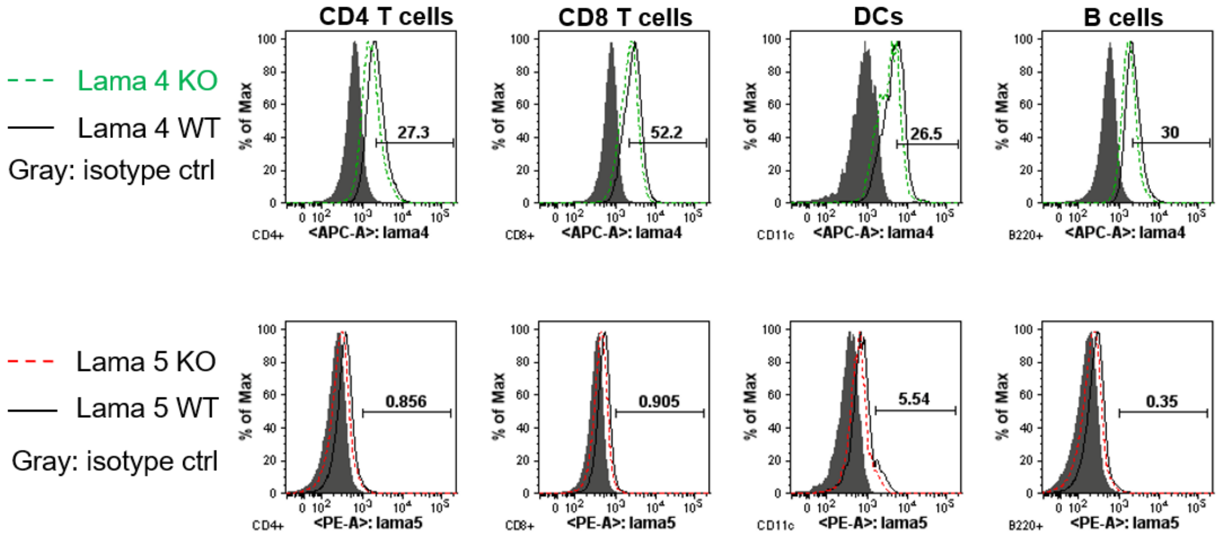

Figure S3. **Lama4 and Lama5 expression in CD4, CD8 T cells, B cells, DC cells**: pLN single cell suspension stained for Lama4 and Lama5, and CD4, CD8, CD11c (DCs) and B220 (B cells). In the graph, values show Lama 4 or Lama 5 percentage.

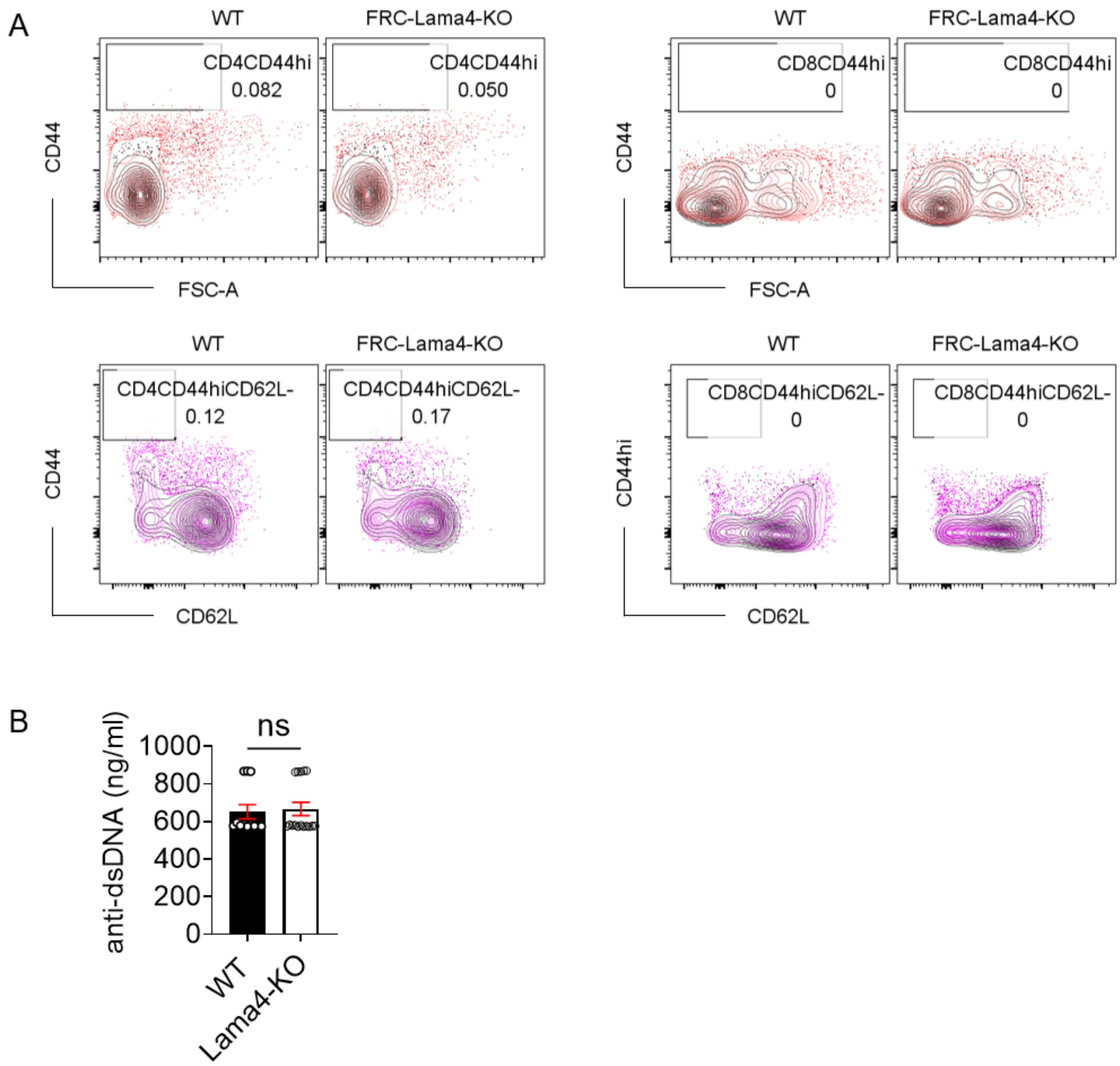

Figure S4. Depleting FRC-Lama4 does not cause systemic cell activation or autoimmunity in naïve or aged mice. A: Assessment of activated T cells in LNs of naive WT and FRC-Lama4-KO mice. Single cell suspensions stained for CD4, CD8, CD44 and CD62L and analyzed using flow cytometry. CD44hi or CD44hiCD62L- populations out of CD4 and CD8 T cells gated as activated cells. Values indicate percentages. **B**: Anti-dsDNA measured in sera of aged WT and FRC-Lama4-KO mice which were over one-year old. Student's unpaired 2-tailed t tests for two groups comparison. Mean ± SEM, n=8, $p < 0.05$ is recognized as significant difference.

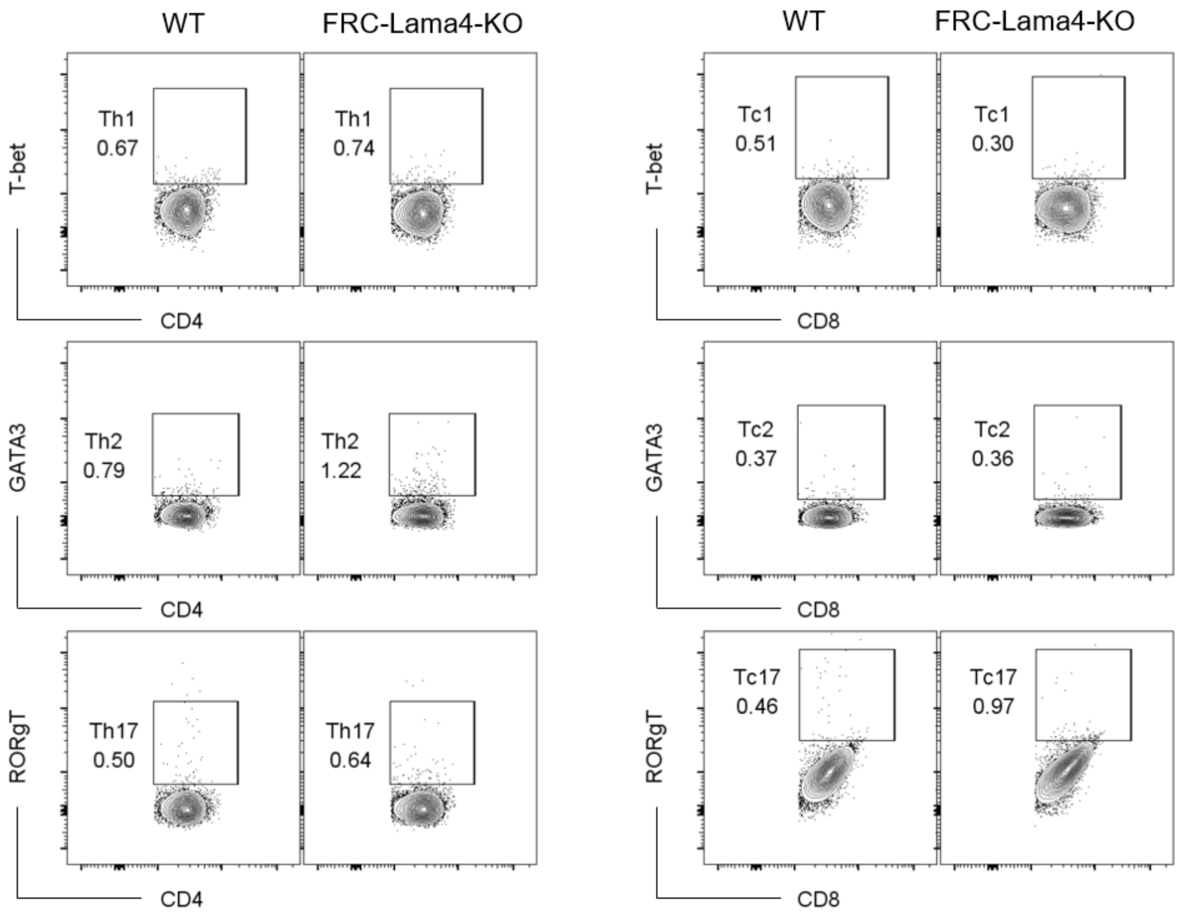

Figure S5. T effector cells in naïve LNs of WT and FRC-Lama4-KO mice. LNs isolated and single cell suspensions stained for CD4, CD8, T-bet, GATA3, RORγT and analyzed using flow cytometry. Values indicate percentage.

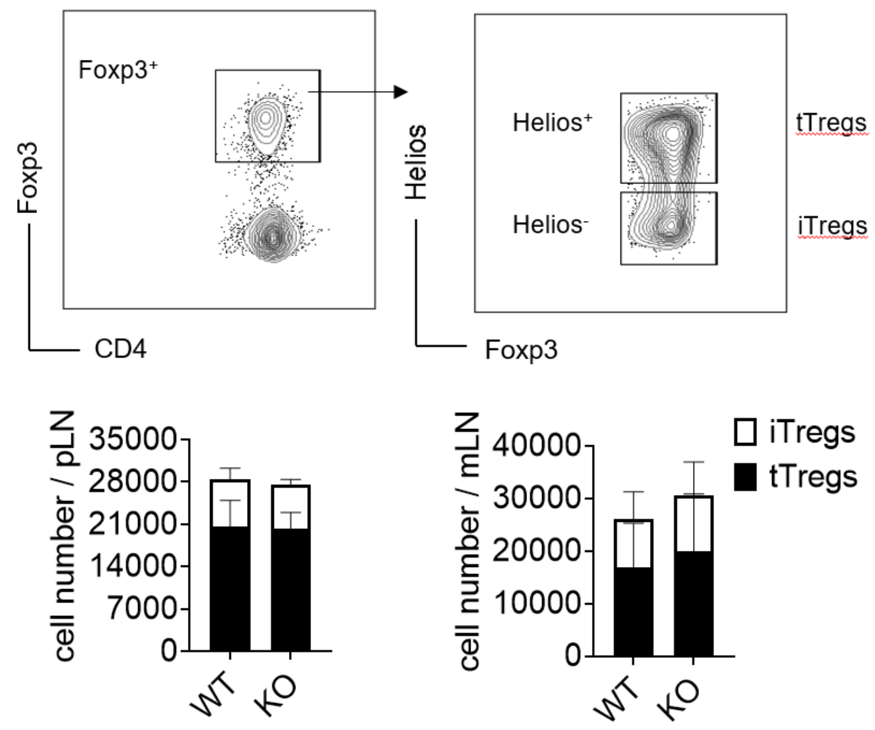

Figure S6. **Thymic Tregs (tTregs, Foxp3$^+$Helios$^+$) and induced Tregs (iTregs, Foxp3$^+$Helios$^-$) abundance in WT and Lama 4 KO LNs**. Gating and quantification of total Tregs (Foxp3$^+$), thymic Tregs (tTregs, Foxp3$^+$Helios$^+$), and induced Tregs (iTregs, Foxp3$^+$Helios$^-$) in Lama4 KO and WT LNs.

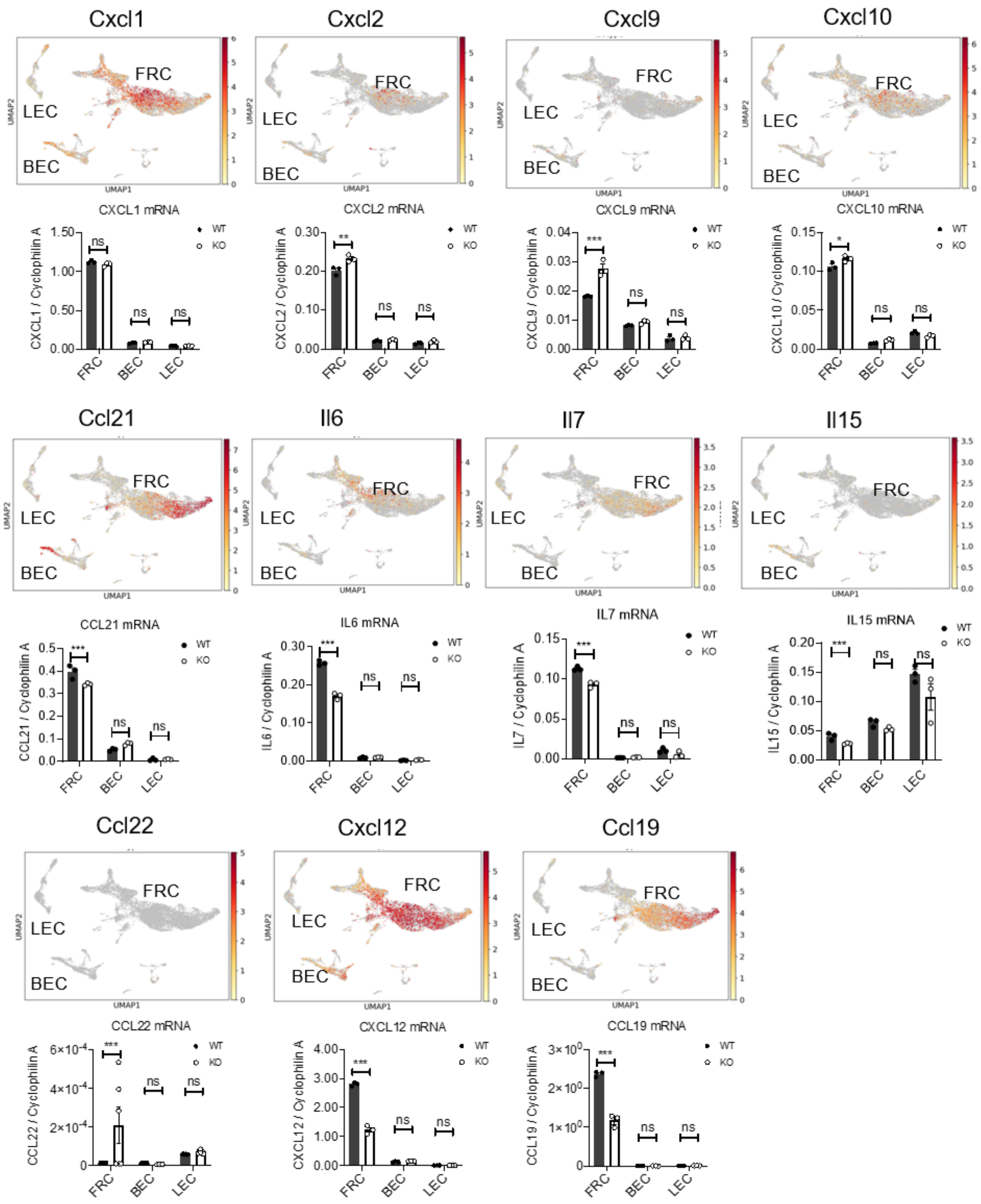

Figure S7. **Depletion of FRC Lama4 alters chemokine and cytokine expressions.** scRNA-seq (feature plots) of WT and qRT-PCR analysis (grouped bar figures) of WT and Lama4 KO mRNA for cytokines and chemokines in LNSC subsets.

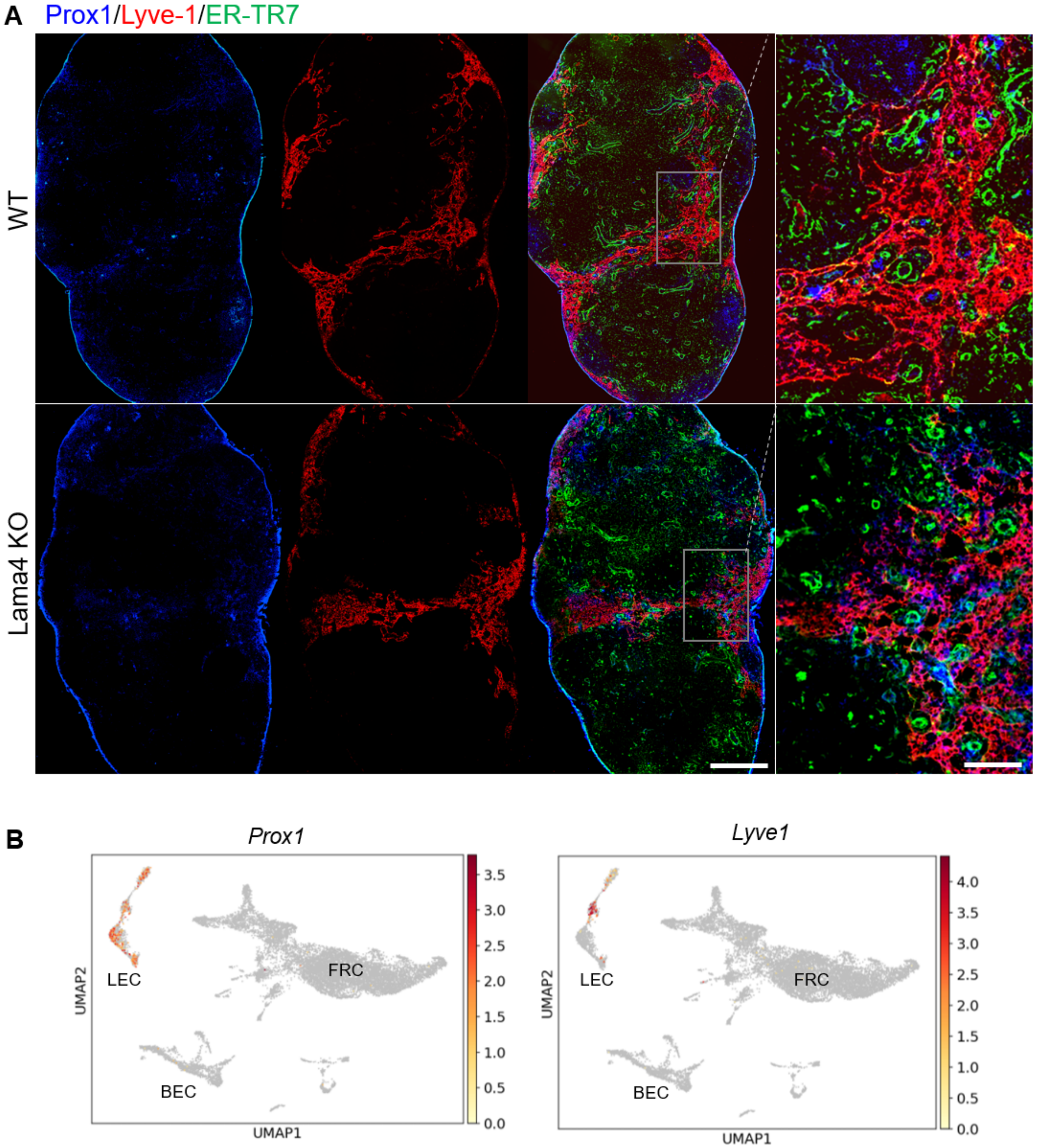

Figure S8. **Depleting Lama4 from LN FRCs does not alter lymphatic vasculature**. **A.** Representative whole mount scanning images of Lama4 KO and WT LN cryosections stained for Prox1, Lyve-1, and ER-TR7. 20x, scale bars, 500 μm (left), 125 μm (right). **B.** scRNA-seq (feature plots) analysis of *Prox1* and *Lyve-1* genes in LNSCs from WT C57BL/6 mice.

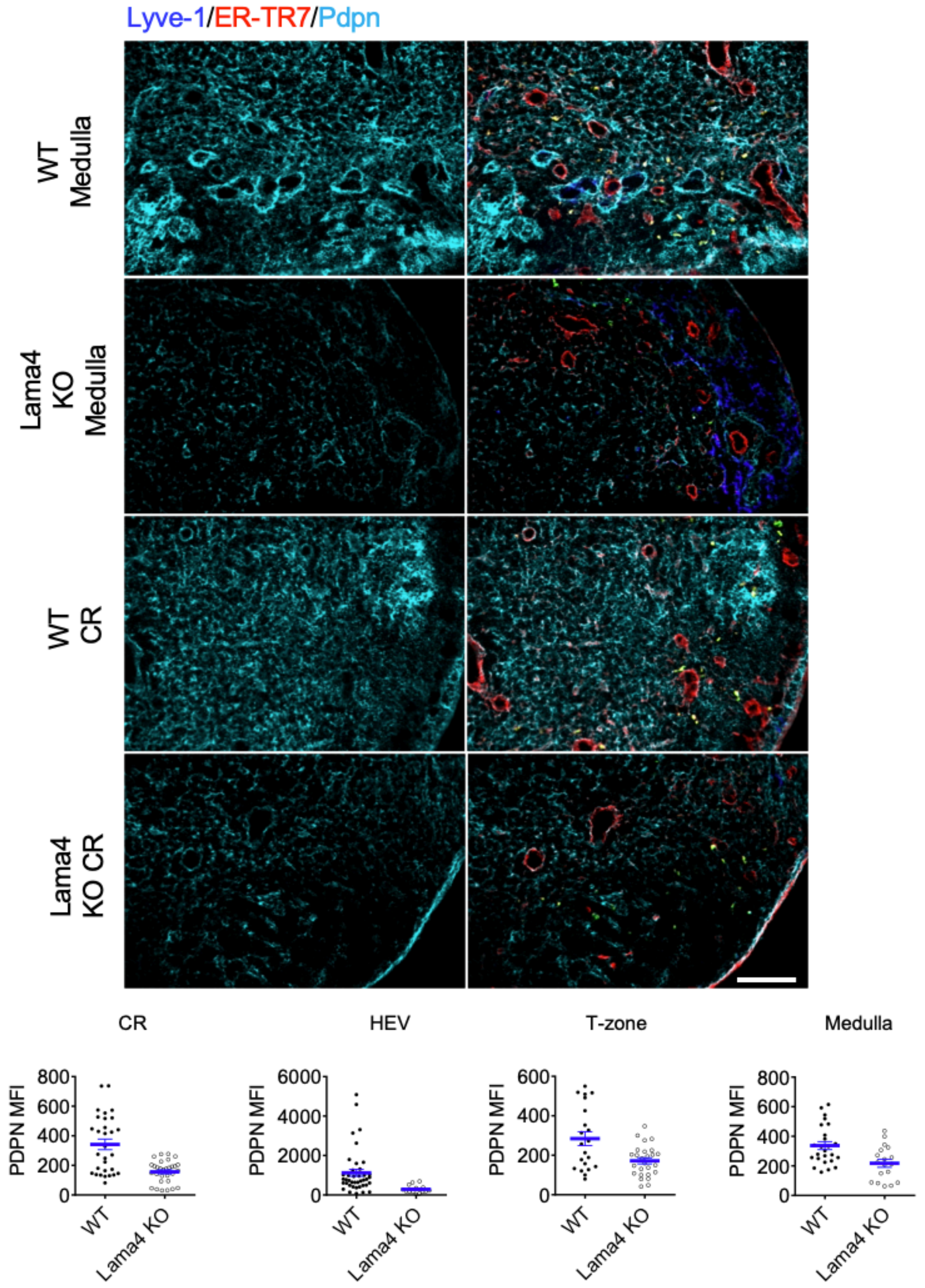

Figure S9. **Depleting FRC Lama4 alters reticular structures.** Representative fluorescent images of Lama4 KO and WT LN cryosections stained for Lyve-1, ER-TR7, and Pdpn. Pdpn quantification in various LN regions. 20x, scale bar, 100 μm. CR: Cortical ridge, HEV: High endothelial venules, T-zone: T cell zone.

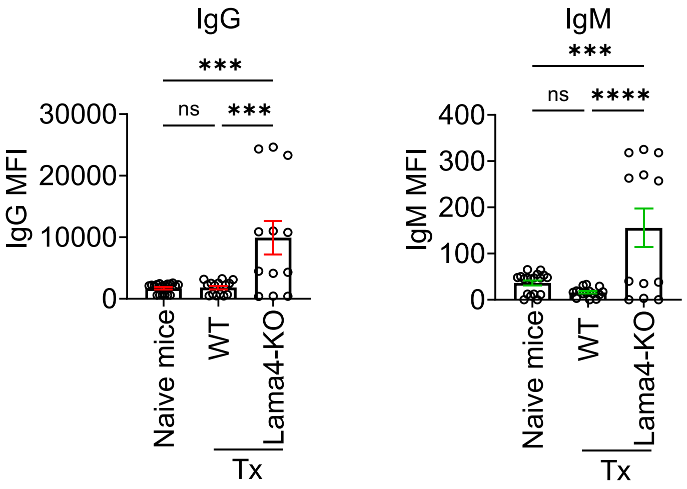

Figure S10. FRC-Lama4-KO recipients produce more alloantibody after cardiac transplantation. WT and FRC-Lama4-KO recipients received BALB/c heart transplants and three doses of anti-CD40L (250ug/dose, i.v., days 0, 4, and 7). Naïve C57BL/6 mice without transplantation served as negative controls. Three weeks after transplantation, allo-antibodies in blood serum measured. n=6, One-way ANOVA with Tukey's multiple comparisons test for multiple groups comparison. Mean ± SEM, ***$p < 0.001$, ****$p < 0.0001$.

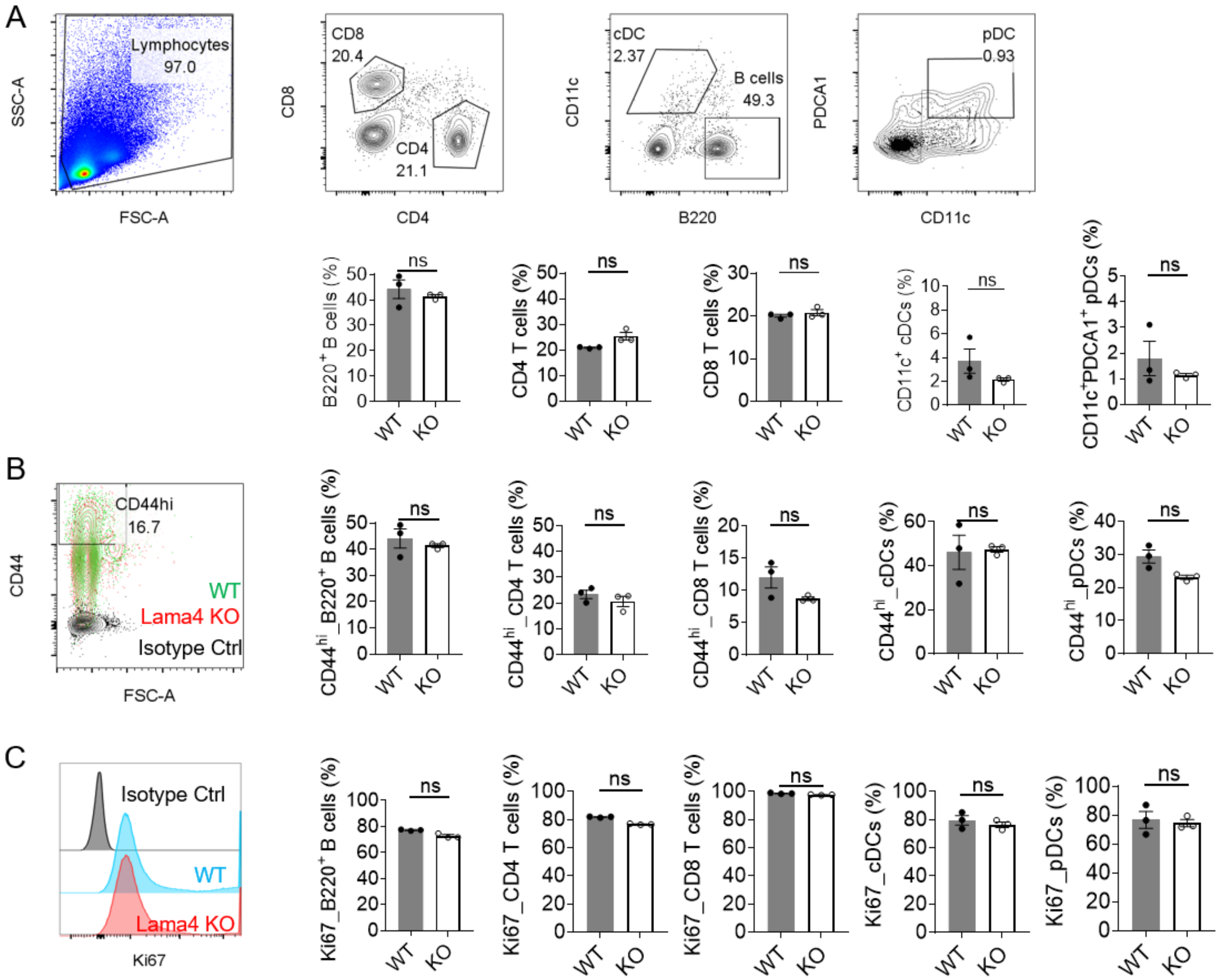

Figure S11. LN immune cell populations, proliferation, and activation in recipients of lung transplants. **A.** 4 days after lung transplantation LNs harvested for analysis of B220^{+} B cells, CD4 T cells, CD8 T cells, CD11c^{+} cDCs and CD11c^{+}PDCA-1^{+} pDCs. **B-C.** CD44 and Ki67 assessed for cell activation and proliferation, respectively.

Supplemental Table 1: Antibodies used for flow and immunohistochemistry

Antibodies	Host	Clone	Application	Vendor
Anti-CD3ε	Rabbit	145-2C11	activation	ThermoFisher Scientific
Anti-CD28	Syrian hamster	37.51	IF	ThermoFisher Scientific
Anti-CD3	Rabbit	Polyclonal	IF	Abcam
Anti-CD3ε	Armenian hamster	145-2c11	activation	ThermoFisher Scientific
Anti-CD4	Rat	GK1.5	FACS	Biolegend
Anti-CD16/32	Rat	93	FACS	ThermoFisher Scientific
Anti-PDCA-1	Rat	927	FACS	Biolegend
Anti-PDCA-1	Rabbit	Polyclonal	IF	FabGennix
Anti-T-bet	Mouse	4B10	FACS	ThermoFisher Scientific
Anti-GATA3	Rat	TWAJ	FACS	ThermoFisher Scientific
Anti-RORγT	Rat	B2D	FACS	ThermoFisher Scientific
Anti-Lama4	Rat	775830	IF	R&D
Anti-Lama5	Rabbit	Polyclonal	IF	Novus Biological
Anti-PNAd	Rat	MECA-79	IF	BD Biosciences
Anti-ER-TR7	Rat	sc-73355	IF	Santa Cruz
Anti-Rabbit IgG	Goat	Polyclonal	IF	Jackson Immunoresearch
Anti-Rat IgG	Goat	Polyclonal	IF	Jackson Immunoresearch
Anti-Rabbit IgG	Donkey	Polyclonal	IF	Jackson Immunoresearch
Alexa Fluor 405 Anti-Rabbit	Donkey	Polyclonal	IF	Jackson Immunoresearch
Alexa Fluor 488 Anti-rat	Donkey	Polyclonal	IF	Jackson Immunoresearch
Alexa Fluor 568 Anti-Goat	Donkey	Polyclonal	IF	Jackson Immunoresearch
Alexa Fluor 647 Anti-rabbit	Donkey	Polyclonal	IF	Jackson Immunoresearch
Anti-CXCL12	Rabbit	Polyclonal	IF	Invitrogen
Anti-CCL21	Rat	AF457	IF	R&D
Anti-CD31	Rabbit	Polyclonal	IF	Abcam
Anti-B220	Rat	RA3-6B2	FACS	ThermoFisher Scientific
Anti-CD45	Rat	S18009F	FACS	Biolegend
Anti-FoxP3	Rat	NRRF-30	IF	Invitrogen
Anti-CD11c	HL3	Armenian Hamster	IF	BD Biosciences

Anti-Lyve1	Rat	ALY7	IF	R&D systems
Anti-IL4	Rat	11B11	Inhibition	ThermoFisher Scientific
Anti-CD69	Armenian hamster	H1.2F3	FACS	ThermoFisher Scientific
Anti-VCAM-1	Mouse	1.4C3	IF	ThermoFisher Scientific
Anti-CD44	Rat	IM7	FACS	ThermoFisher Scientific
Anti-Foxp3	Rat	PCH101	FACS	ThermoFisher Scientific
Anti-CD40L	Armenian Hamster	MR-1	Inhibition	Bio X Cell
Anti-IL-33	Goat	Polyclonal	IF	R&D
Anti-CD62L	Rat	MEL-14	Inhibition	BioXcell
Anti-Pdpn	Syrian hamster	eBio8.1.1	FACS	ThermoFisher Scientific
Anti-IgM	Rat	II/41	FACS	ThermoFisher Scientific
Anti-IgG	Goat	Poly4053	FACS	Biolegend

Supplemental Table 2: Primer sequences for RT-PCR

Target	Forward	Reverse
**Lama5**	5'-GGACCTCTACTGCAAGCTGGT-3'	5'-ATAGGCCACATGGAACACCTG-3'
**Lama4**	5'-AAGCCTCAAGAAAGGGTATGC-3'	5'-AAATGTTGCCCTATGGCTTG-3'
**CXCL1**	5'-GGGCGCCTATCGCCAAT-3'	5'-ACCTTCAAGCTCTGGATGTTCTTG-3'
**CXCL2**	5'-GAAGTCATAGCCACTCTCAAGG-3'	5'-CTTCGGTTGAGGGACAGC-3'
**CXCL9**	5'-AATGCACGATGCTCCTGCA-3'	5'-AGGTCTTTGAGGGATTTGTAGTGG-3'
**CXCL10**	5'-GCCGTCATTTTCTGCCTCA-3'	5'-CGTCCTTGCGAGAGGGATC-3'
**CXCL11**	5'-ATGGCAGAGATCGAGAAAGC-3'	5'-TGCATTATGAGGCGAGCTTG-3'
**CCL19**	5'-ATGCGGAAGACTGCTGCC-3'	5'-CGGAAGGCTTTCACGATGTT-3'
**CCL21**	5'-TCCCGGCAATCCTGTTCTT-3'	5'-CCTTCCTCAGGGTTTGCACA-3'
**CCL22**	5'-TCCCTATGGTGCCAATGTG-3'	5'-ATATCTCGGTTCTTGACGGTTATC-3'
**IL-6**	5'-GAGGATACCACTCCCAACAGACC-3'	5'-AAGTGATCATCGTTGTTCATACA-3'
**IL-7**	5'-GATAGTAATTGCCCGAATAATGAACCA-3'	5'-GTTTGTGTGCCTTGTGATACTGTTAG-3'
**IL-15**	5'-CCATCTCGTGCTACTTGTG-3'	5'-CTGTTTGCAAGGTAGAGCACG-3'
**CXCL12**	5'-CTCTGCATCAGTGACGGTAA-3'	5'-CTTCAGCCGTGCAACAATCT-3'
**VCAM-1**	5'-GCAGGATGCCGGCATATACG-3'	5'-TGCGCAGTAGAGTGCAAGGA-3'
**ICAM-1**	5'-ACCCCAAGGACCCCAAGGAGAT-3'	5'-CGACGCCGCTCAGAAGAACCA-3'
**MAdCAM-1**	5'-AGAAGAGGAGATACAAGAGG-3'	5'-TAGTGTCTGGGCGAGGACC-3'
**VEGF-A**	5'- TTACTGCTGTACCTCCACC-3'	5'- ACAGGACGGCTTGAAGATG-3'
**VEGF-C**	5'-ACCGTGTGCGAATCGACTG-3'	5'-AATACGATGGGACACAGCGG-3'
**VEGF-D**	5'- TTGACCTAGTGTCATGGTAAAGC-3'	5'- TCAGTGAACTGGGGAATCAC-3'
**Cyclophilin A**	5'-AGGGTGGTGACTTTACACGC-3'	5'-ATCCAGCCATTCAGTCTTGG-3'
